# Exploring the importance of predisposing, enabling, and need factors for promoting Veteran engagement in mental health therapy for post-traumatic stress: a multiple methods study

**DOI:** 10.1186/s12888-023-04840-7

**Published:** 2023-05-27

**Authors:** Megan Shepherd-Banigan, Abigail Shapiro, Karen M. Stechuchak, Kate L. Sheahan, Princess E. Ackland, Valerie A. Smith, Barbara G. Bokhour, Shirley M. Glynn, Patrick S. Calhoun, David Edelman, Hollis J. Weidenbacher, Madeleine R. Eldridge, Courtney H. Van Houtven

**Affiliations:** 1 Durham VA Health Care System, 508 Fulton Street, Durham, NC 27705 USA; 2 Department of Population Health Sciences, Duke University School of Medicine, 215 Morris Street, Durham, NC 27701 USA; 3 Duke-Margolis Center for Health Policy, Box 90120, 100 Fuqua Drive, Durham, NC 27708 USA; 4 Center for Care Delivery and Outcomes Research, Minneapolis VA Health Care System, One Veterans Drive, Minneapolis, MN 55417 USA; 5 Department of Medicine, University of Minnesota, 420 Delaware St SE, Minneapolis, MN 55455 USA; 6 Division of General Internal Medicine, Department of Medicine, Duke University School of Medicine, 200 Morris Street, Durham, NC 27701 USA; 7 Center for Healthcare Organization and Implementation Research, VA Bedford Healthcare System, 200 Springs Road (152), Bedford, MA 01730 USA; 8 Department of Population and Quantitative Health Sciences, University of Massachusetts Chan Medical School, 368 Plantation Street, The Albert Sherman Center, Worcester, MA 01605 USA; 9 UCLA Semel Institute of Neuroscience and Human Behavior, VA Greater Los Angeles Healthcare System at West Los Angeles, B151 11301 Whiltshire Boulevard, Los Angeles, CA 90073 USA; 10 Department of Psychiatry & Behavioral Sciences, Duke University School of Medicine, 905 West Main Street, Durham, NC 27701 USA

**Keywords:** Multiple methods, PTSD, Care utilization, Family support

## Abstract

**Purpose:**

This study explored Veteran and family member perspectives on factors that drive post-traumatic stress disorder (PTSD) therapy engagement within constructs of the Andersen model of behavioral health service utilization. Despite efforts by the Department of Veterans Affairs (VA) to increase mental health care access, the proportion of Veterans with PTSD who engage in PTSD therapy remains low. Support for therapy from family members and friends could improve Veteran therapy use.

**Methods:**

We applied a multiple methods approach using data from VA administrative data and semi-structured individual interviews with Veterans and their support partners who applied to the VA Caregiver Support Program. We integrated findings from a machine learning analysis of quantitative data with findings from a qualitative analysis of the semi-structured interviews.

**Results:**

In quantitative models, Veteran medical need for health care use most influenced treatment initiation and retention. However, qualitative data suggested mental health symptoms combined with positive Veteran and support partner treatment attitudes motivated treatment engagement. Veterans indicated their motivation to seek treatment increased when family members perceived treatment to be of high value. Veterans who experienced poor continuity of VA care, group, and virtual treatment modalities expressed less care satisfaction. Prior marital therapy use emerged as a potentially new facilitator of PTSD treatment engagement that warrants more exploration.

**Conclusions:**

Our multiple methods findings represent Veteran and support partner perspectives and show that amid Veteran and organizational barriers to care, attitudes and support of family members and friends still matter. Family-oriented services and intervention could be a gateway to increase Veteran PTSD therapy engagement.

**Supplementary Information:**

The online version contains supplementary material available at 10.1186/s12888-023-04840-7.

## Background

Post-traumatic stress disorder (PTSD) is a disabling and costly psychiatric disorder estimated to occur in 23% of Veterans with recent service in Iraq and/or Afghanistan [11]. PTSD symptoms are related to problems with work role [10] and family functioning [37]. To improve treatment for Veterans with PTSD, the Department of Veterans Affairs (VA) has disseminated evidence based psychotherapies for PTSD nationally [8], yet Veteran uptake of these therapies is persistently low. Across studies, rates of PTSD therapy initiation range from 24 to 50% [9, 15, 18, 41] while rates of receipt of an adequate dose range from 24 to 52% [9, 18, 41].

**Fig. 1:**
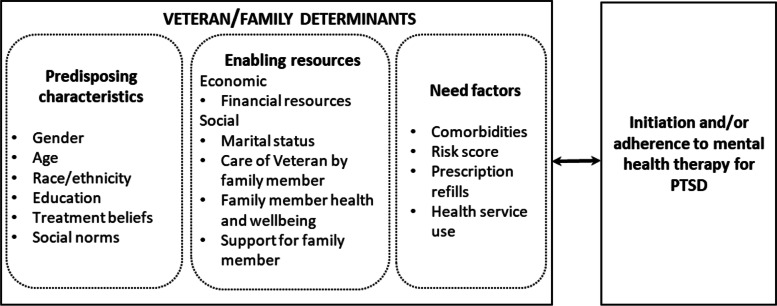
Conceptual model adapted from Andersen’s Behavioral Model of Health Care Access

Efforts to understand lack of PTSD treatment engagement have focused on predictors of initiation of and retention in therapy, including age, gender, behavioral health comorbidities [19, 25, 33]. The Andersen behavioral model of health service utilization provides a useful theory to conceptualize predictors of PTSD therapy use (Fig. 1) [1, 19]. This model posits that health service use is a complex function of predisposing characteristics, enabling resources, and need factors. Predisposing characteristics include age, gender, treatment attitudes, and social norms. Enabling resources include economic resources, such as money for transportation; and social resources, such as social support for treatment. Need factors are health conditions that require health care [1].

The majority of extant evidence has focused on the relationship between Veteran pre-disposing and need factors and PTSD therapy engagement [19, 26, 30, 39]. Less literature has focused on enabling factors, but suggests that social support could be important for treatment engagement [42]. Economic constraints that could lead to logistical barriers to treatment like not having financial resources to get to appointments [4] have not been considered previously to our knowledge.

### Contribution to the field

Engagement in PTSD treatment is an extremely complex behavior and is likely a function of multiple factors that cannot be assessed using a single data source or through patient perspectives only. As a result, the findings from existing literature about the most critical determinants of PTSD treatment engagement are mixed. In this study, we aim to advance our understanding of determinants of PTSD therapy use by expanding the number of factors considered together using information derived from both quantitative and qualitative data to draw a more complete picture. We also incorporated perspectives of both Veterans and their family/friends to capture information about social contexts and their impact on treatment engagement.

## Methods

### Study purpose

The purpose of this study is to advance a contextually rich understanding of how predisposing, enabling, and need factors promote Veteran engagement in PTSD therapy using a multiple perspective and methods approach. Our research questions are:

1. Which predisposing, enabling, or need factors are the most influential drivers of Veteran PTSD therapy initiation and completion of an adequate dose (or treatment retention)?
2. In what ways do Veterans and their family members perceive predisposing, enabling and need factors to promote engagement in PTSD therapy?

### Methods overview

Given the lack of clear justification from past studies and theory about which enabling factors are most important for PTSD care-seeking, we apply a machine learning approach, which allows for data-driven exploration in situations where there is limited information to inform hypotheses. We applied this method to a quantitative dataset that includes a rich set of predisposing, enabling, and need factors to learn which factors most influence *both* Veteran initiation and retention to PTSD therapy. We also considered economic resources as an explicit enabling factor. In this way, we can elucidate how these factors operate within the constellation of other drivers of treatment engagement. Second, the role of economic and social enabling factors to promote care-seeking for mental health are promising, as are family-level or household constructs. Yet the perspectives of family members are rarely captured, and existing knowledge is primarily based on Veteran perspectives. We include quantitative data collected from family members and friends who we refer to as “support partners”. Third, as our quantitative dataset did not capture factors including treatment attitudes, social support, and social norms, we supplemented this data with qualitative data gathered from Veterans and their support partners to explore how participants talk about predisposing, enabling, and need factors to augment what we learned from the quantitative data. All study activities were reviewed and approved by the Durham VA Institutional Review Board (Protocol #02227).

### Study design

We applied a multiple methods design to enhance our understanding of determinants of treatment engagement and overcome the limitations of quantitative- and qualitative-only approaches. For example, our quantitative approach generates insights from a large sample with many potential determinants of treatment engagement, but its reductionist approach does little to elucidate nuances in how these determinants function [17]. While our qualitative analysis provides a nuanced understanding built on Veteran and family member perspectives, we are limited in the number of constructs we could explore. Our quantitative study is a secondary analysis of VA medical record data from an existing cohort of Veterans [49] merged with survey data from an associated support partner (e.g., partner, spouse). This dataset was originally used to evaluate the VA Program for Comprehensive Assistance for Family Caregivers (PCAFC) [49]. While this choice limits our sample to Veterans/supports partners who enrolled in PCAFC, this dataset includes a rich set of support partner reported information matched to the medical records of the Veterans for whom they provided care that we would have been unable to collect otherwise because support partners are not systematically identified in VA medical records [29]. Furthermore, the dataset included information about predisposing, enabling, and need factors and sample size needed to address the research question. We analyzed the quantitative data using machine learning algorithms to explore which drivers were most strongly related to initiating a new mental health treatment episode and completing an adequate dose, defined as at least 8 sessions [41]. In parallel, we interviewed 18 Veterans and 13 associated support partners about factors that promoted or hindered engaging in PTSD mental health therapy to provide more breadth and depth for understanding the quantitative results [22]. Results for both studies were analyzed separately according to constructs of the Andersen model. Study procedures were approved by the Durham VA Institutional Review Board (Protocol #02227). Table 1 provides an overview of the design for the quantitative and qualitative studies.

**Table 1:** Overview of qualitative and quantitative arms of the study

	**Goal of study**	**Sampling Strategy**	**Sample Size**	**Timeframe of data collection**	**Data Sources**	**Analytical Approach**
***Quantitative***	To identify the most important predisposing, enabling, and need factors that predict PTSD treatment	Veterans with PTSD diagnosed between Jan 1, 2000 and prior to the survey who were enrolled in the VA Caregiver Support Program with a support partner between May 1, 2010 and September 1, 2015	1,047 Veterans and support partner dyads	EHR baseline variables assessed May 1, 2011- September 1, 2015 Survey data collected September–October 2015 Outcomes assessed December 1, 2015 – September 30, 217	Veteran health record data Caregiver Support Program administrative data Survey completed by support partners	Random forest
***Qualitative***	To provide a deeper understanding of predisposing, enabling, and need factors captured and not captured in structured data that are important to Veterans and support partners	Veterans referred for PTSD therapy within 18 months window prior to qualitative study recruitment (December 2019) who applied to the VA Caregiver Support Program with a support partner between May 1, 2010 and May 7, 2019	18 Veterans 13 support partners Data from 30 participants was included (see Fig. 1) (Out of 31 participants, 11 dyads participated)	Data collected February 2020-February 2021	Semi-structured interviews with Veterans and support partners (conducted separately)	Rapid analysis of themes

### Quantitative procedures

#### Sample

Quantitative data are from a prior study of dyads of Veterans and an associated support partner who had applied to the VA Program for Comprehensive Assistance for Family Caregivers (PCAFC) between May 1, 2010 and September 1, 2015 and subsequently enrolled in the program for at least 90 days (REFERENCE BLINDED FOR REVIEW). PCAFC is a national program of the VA Caregiver Support Program and supports eligible Veteran VA users who served during the Iraq and Afghanistan conflicts and were injured during military service. The support partners in our sample had completed a self-administered, web-based survey in September or October 2015 (*n* = 1,407); recruitment rate was 14%, and the sampling process is briefly described in the Additional file 1: Methodological Appendix 1.1. Full details about the survey methodology and cohort are published elsewhere (REFERENCE BLINDED FOR REVIEW). The sample for the present study was constrained to dyads for whom Veterans had an International Classification of Disease, 9^th^ Revision (ICD-9) diagnosis for PTSD in their VA medical records anytime between January 1, 2000 and September 2, 2015 and who had not attended a PTSD psychotherapy visit during the baseline period defined as September 4, 2014 and September 3, 2015; 170 dyads were removed due to these exclusion criteria yielding an analytical sample of *n* = 1,237 dyads.

#### Data sources

The existing dataset had merged information from three data sources, including VA electronic health records, administrative data from the Caregiver Support Program, and survey data from support partners of Veterans. Administrative data sources included variables such as drive time to the nearest VA, PTSD medication refill, Veteran healthcare need risk score (called Nosos score), and Veteran diagnoses. Additional file 2: Table S1 provides more information on the data source of each variable.

#### Survey

The survey included 100 questions about support partner and Veteran socio-demographics, emotional, health, and financial wellbeing (e.g., level of education, income, depressive symptoms, health status, perceived financial stress), caregiving experiences (e.g., time spent caregiving, subjective burden, positive attitudes towards caregiving), and use of and satisfaction with services offered through the PCAFC. The survey used validated measures when possible, including the Center for Epidemiological Studies-Depression (CESD-10) scale for depressive symptoms [2], the Zarit subjective burden scale for support partner burden [3], a subscale of the Caregiver Reaction Assessment for perceived financial strain [13], Positive Aspects of Caregiving [44], and VR-12 health status [23].

#### Outcomes

The main outcomes of interest were Veteran initiation of and retention in an episode of VA-provided mental health therapy for PTSD. Mental health therapy visits were identified using clinic visit codes, provider-classification, and ICD-10 codes that indicated that the Veteran received individual or group behavioral counseling for PTSD (Spoont, personal communication 12/6/19) (Additional file 1: Methodological Appendix 2.1). If a Veteran had more than one qualifying visit on the same day, we counted those visits as one single visit. Initiation of a treatment episode was defined as at least two sessions of therapy received on different days occurring within 21 days of one another between December 1, 2015 and September 30, 2017 (determined via conversations with VA clinicians and researchers who were part of project Advisory Board 12/10/19). We designated two visits to maximize the possibility that Veterans engaged in therapy because the first visit could indicate an evaluation visit and not actual therapy and we limited the space between treatment to 21 days as visits that occur 30 days apart might indicate case management (Spoont, personal communication 12/6/19). We used the same definition as prior studies for completion of an adequate dose of treatment (referred to as “retention”); this was specified as the receipt of at least 8 sessions of therapy [41] received within 180 days between December 1, 2015 and September 30, 2017.

#### Treatment drivers

We modeled 55 treatment drivers that aligned with constructs in the Andersen model (e.g., pre-disposing, enabling, and need). The variables are presented in in Additional file 2: Table S1 and are organized by model constructs and presented as they were specified in the models. Briefly, pre-disposing factors included age, gender, marital status, etc.; enabling resources included marital status, financial strain, family member wellbeing, etc.; and need factors included health service use, medical diagnoses, Nosos (i.e., risk score denoting general need for health services), etc. Baseline was defined as the date the survey was deployed (September 3, 2015).

#### Quantitative data analysis

The quantitative analysis aimed to identify the most influential Veteran and family-level drivers of Veteran initiation of and retention in mental health therapy for PTSD. This study is exploratory in that there are not well-defined evidence-based or theoretical a priori hypotheses about which drivers are most influential, especially when we consider information reported by support partners. Machine learning algorithms search for patterns in the data and can identify complex patterns to support new insights and develop hypotheses. We applied well-known algorithms and accepted measures of rigor specific to machine learning approaches which include training the algorithms (objectivity), reporting predictive fit (validity), and assessing the consistency (reliability) of our model results; for details see Additional file 1: Methodological Appendix 3. We used a classification random forest algorithm for binary outcomes [5]. Random forests identify the relative importance of variables in the model and do not produce estimates of effect or assign direction of effects.

We created a dataset for each outcome. Missing data were imputed for each dataset using a machine learning algorithm to predict missing values [21]. We applied a sampling rebalancing technique to the retention outcome dataset [40]. Using accepted convention, we split each outcome dataset into a 70% training dataset and 30% testing dataset. For each outcome, we first tuned the parameters in the training dataset, we next ran the best fitting model in the test dataset to assess how predictive this model was, and then ran the best model in the full dataset to estimate the relative importance of each variable in driving the outcomes [27]. Random forests were estimated using 1,000 bootstrapped trees. To select the most influential variables presented in this paper, we ran the outcome models using different specifications of the random forest algorithm and then two analysts compared the results across algorithms to identify the most influential variables that appeared consistently. We estimated the bivariate association of these variables using regression models to understand the direction of effect. We conducted a robustness check using cross-fold validation to assess the consistency of the results of our primary model. RStudio version 4.0.2 and SAS version 9.4 were used. See Additional file 1: Methodological Appendix 4.1 for details.

### Qualitative procedures

#### Participants and data sources

**Fig. 2:**
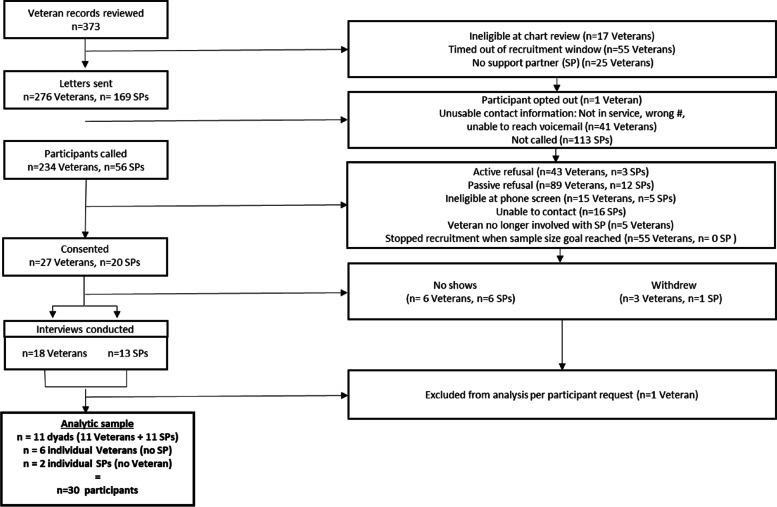
Qualitative Study flow. Note: SP = support partner; NIS = number not in service

Interviews were conducted with a parallel sample recruited for this study [7]. Qualitative participants were identified from Veterans Health Administration administrative data. Veterans had an ICD-10 PTSD diagnosis and had applied to PCAFC. The target support partner was the individual who was associated with the most recent application to PCAFC between May 1, 2010 and May 7, 2019. In one case the support partner participant was not the person who applied to PCAFC but was the Veteran’s spouse. The unit of observation was the dyad regardless of whether both individuals enrolled in the study. To qualify, Veterans also had to have a referral for a VA mental health visit in the 18 months prior to the data pull in December 2019, to recall receiving that referral, and to report being in touch with the support partner who applied with them to PCAFC. Eligible Veterans were randomly sorted to receive letters with study details and an opt-out number. If potential participants did not opt-out within 7 days, participants were contacted via telephone by study staff to explain the purpose of the research, convey the risks and benefits of study participation, and to assess eligibility. Once the study team enrolled the Veteran, they attempted to enroll the support partner using the same process. Participants enrolled in the study provided verbal consent to participate in the study and received $25 for completing the interview. See Fig. 2 Qualitative Study Flow.

#### Data collection

Interview guides were developed to understand barriers and facilitators to engaging in PTSD therapy currently and in the past and were modified after an initial review of the first set of interviews. Veterans were asked about predisposing factors (e.g., treatment attitudes, past treatment experiences, motivations, social norms, enabling factors (e.g., economic and social facilitators and barriers to treatment initiation and engagement), and need factors (e.g., descriptions of mental health needs, desired outcomes for treatment). Veterans were also asked about social support they received for their mental health from all sources and from their named support partner from the PCAFC application and the role of social support for treatment. Support partners were asked to describe their understanding of and perspective on the Veteran’s mental health treatment and how involved they were in the Veteran’s past and current mental health treatment episodes.

Data were collected between February 2020 and February 2021 by trained interviewers (AS, MSB, HW); participant recruitment paused between April-August 2020 due to challenges related to the COVID-19 pandemic. Interviews (*n* = 31) were semi-structured and lasted approximately 30 min. Veterans (*n* = 18) and support partners (*n* = 13) were interviewed separately. Interviews were digitally recorded and transcribed (*n* = 26) or documented through detailed notes (*n* = 5). One participant requested that their data not be analyzed and so the analytical sample is *n* = 30.

#### Qualitative data analysis

Transcripts were reviewed and summarized. We used Hamilton’s rapid analysis approach to structure the qualitative inquiry [16]. The qualitative analyst (AS) created a summary template for each dyad (or individual if only one member of the dyad was interviewed) that identified barriers and facilitators of treatment engagement organized by the main constructs in the Andersen model (e.g., pre-disposing, enabling, need). One of three members of the qualitative team summarized each transcript/interview notes into this template and another member reviewed each summary for accuracy. To establish credibility, the team met weekly to discuss “similarities within and across study participants” and emerging themes and their reactions to the data [45]. Then, the data from each summary template was transferred into a single matrix and coders summarized the information by construct across dyads/cases with each construct reviewed by two coders. To establish dependability and confirmability, the coding team met to compare summaries and then met again to review all summaries, identify emerging themes, and to discuss their interpretation of the data [45]. Once the team developed written narrative of the themes, data in the matrices and transcripts were reviewed again to ensure that themes reflected participants’ reporting.

### Comparing qualitative and quantitative findings

Once the qualitative and quantitative data were analyzed, the coders applied a deductive approach that overlaid insights from the qualitative data (on therapy engagement) and findings from the quantitative data (on therapy initiation and retention) to the Andersen model constructs. To do this, the coding team compared findings that emerged from the quantitative analysis to themes from the qualitative analysis to identify how quantitative findings were better understood by what participants reported during the interviews. We supplemented this analysis with quotes from the transcripts to illustrate participants’ perspectives. We then compared findings across data sources to identify common themes and where findings diverged. We developed overarching narrative summaries of results that incorporated insights from both the qualitative and quantitative data. Finally, we reread the transcripts to ensure that the integrated findings aligned with the interview data.

## Results

### Quantitative results

Table 2 shows demographic characteristics of the quantitative cohort.

**Table 2:** Descriptive statistics for quantitative cohort

**Characteristic**	Veteran (*n* = 1,237)	Support partner (*n* = 1,237)
Male sex^a^, n (%)	1156 (93.5)	67 (5.4)
Age^a^, mean (SD)	40.9 (9.4)	42.8 (11.9)
Race^a^		
White, n (%)	794 (65.3)	744 (64.6)
Black, n (%)	236 (19.4)	182 (15.8)
Other^b^, n (%)	186 (15.3)	225 (19.5)
Hispanic/Latino(a) ethnicity^a^, n (%)	294 (23.9)	294 (24.3)
Veteran and family member partnered^a^, n (%)	1061 (86.1)	-

^a^Missing data: support partner age (131), support partner gender (2), Veteran race (21), support partner race (86), Veteran Hispanic/Latino(a) ethnicity (7), support partner Hispanic/Latino(a) ethnicity (26), Veteran and support partner partnered (4)

^b^Includes Asian, American Indian or Alaska Native, Native Hawaiian or Other Pacific Islander, or multiple racial categories

#### Quantitative model robustness

In the test set, the treatment initiation and retention models yielded area under the curve (AUC) values of 0.59 and 0.84, respectively, indicating that the treatment initiation models were less accurate. An AUC of 0.5 indicates that the model is assigning the correct outcome to 50% of the cases which suggests that the model has no discriminate ability whereas an AUC of 1.0 indicates that the model is correctly assigning the outcome in 100% of the observations. When examining how consistent the results were across five randomly constructed subsamples of the data, for treatment initiation the top ranked variable was identified in one tree, but other trees identified no splits. For treatment retention, the top ranked variables were selected in all 5 subsamples.

#### Drivers of initiation of PTSD therapy

Greater need for health care services was by far the most important driver of initiation of PTSD therapy across the random forest models. Concurrent Nosos score for FY2015 [50], which is a single variable risk score that denotes risk for higher VA healthcare costs compared to average VA users, was used as one proxy for general need of health services in the analytical models. This risk score comprises health service use costs, prior utilization, physical and mental health diagnoses, prescription use, and demographic data. The Nosos risk score is an inclusive indication of need for services because it addresses costs, use, and diagnoses especially so within the VA which as an integrated health care system is incentivized to align need with use and costs as much as possible.

#### Drivers of retention in PTSD therapy

Highly ranked variables in our main random forest model that were also identified in the secondary models were primarily social enabling and need variables; these included: 1) not being married, 2) family therapy visit in the 12 months prior to the survey, 3) substance use diagnosis in the past 12 months, 4) VA emergency department visit in the past 12 months, 5) number of PTSD medication refills in the past 12 months, and 6) higher Nosos score.

### Qualitative findings

Table 3 shows the demographic characteristics of the qualitative sample.

**Table 3:** Descriptive statistics for qualitative sample participants (*n* = 31)

**Characteristic**		
	**Veterans (*n* = 18)**	**Support partners (*n* = 13)**
**Age, median (minimum – maximum)**	46 (31–57)	41 (28–72)
	**n (%)**	
**Sex (self-reported)**		
Male	9 (50.0)	5 (38.5)
Female	9 (50.0)	8 (61.5)
**Race (self-reported)**		
White/Caucasian	5 (27.8)	4 (30.8)
Black/African American	5 (27.8)	5 (38.5)
Asian	1 (5.6)	0 (0.0)
Native American/Pacific Islander	3 (16.7)	0 (0.0)
Mixed	2 (11.1)	2 (15.4)
Other	1 (5.5)	2 (15.4)
Prefer not to answer	1 (5.5)	0 (0.0)
**Ethnicity (self-reported)**		
Hispanic, Latino(a)	3 (16.7)	5 (38.5)
**Veteran/Support partner Relationship**	**Dyads**^**a**^ ***n* = 11**	
Spouse	8 (72.7)	
Parent/Child	1 (9.1)	
Significant Other	2 (27.3)	

^a^Both Veteran and caregiver in that dyad were interviewed

#### Predisposing factors

Treatment attitudes emerged as an important predisposing factor for treatment engagement in the interviews. Positive treatment attitudes stemming from perceived treatment benefits underpinned Veterans’ motivation to engage in treatment. As one Veteran who recently completed an episode of therapy noted,

“I loved that [10-week evidence-based therapy program]. … I worked on doing my homework, that was the best thing I’ve completed. I learned how to cope with a lot and what to do and realizing that it’s never going to go away. I recognized what my triggers were. Faithfully going to those appointments and practicing what we were working on was amazing” (Veteran).

Some support partners also described positive attitudes towards treatment because they believed treatment would be helpful and was needed for the Veteran’s symptoms to improve. The spouse of the Veteran quoted above had urged the Veteran to seek mental health care at the VA because the Veteran had become withdrawn and irritable. The Veteran went on to be diagnosed with PTSD and through therapy she was able to manage her symptoms: “It helped with her moods, her general attitude on daily life, feeling better about herself. She doesn’t see it [changes in symptoms] all the time, but there is a difference” (Support Partner).

Negative treatment attitudes, including those influenced by past negative experiences with treatment (i.e., PTSD symptom exacerbation, structural barriers), concerns about how difficult the experience of treatment would be, or beliefs that the benefits of treatment would be minimal, reduced motivation to seek a new course of treatment. One Veteran described how talking about his trauma in therapy made him feel worse in the moment, “Just it’s rough talking about it already…because going in there, you’re fine. But when you start getting into your emotions and what’s going on in your brain, it just makes it a whole lot worse” (Veteran). He declined future referrals for PTSD treatment.

Motivation to engage in therapy was also tied to social relationships in that several Veterans expressed that they were seeking a way to manage their symptoms to improve interactions with support partners. One Veteran said, “Well, the most important was to improve my marriage. My marriage was on the brink, my family, my children. It improved that, and that was important to me” (male Veteran). Positive family perceptions about the value of treatment further bolstered the Veteran’s motivation to seek treatment. One Veteran mentioned that when her spouse noticed that she is having a hard time, he encouraged her to go to therapy and her reaction was, “You’re right. I do need to get seen. I am having a rough time” (Veteran).

The impact of other predisposing factors on treatment engagement did not come up during the interviews, with the exception of gender and age. For example, we found that gender might be a barrier to care in that Veterans with specific conditions, such as military sexual traumas or those (i.e., female Veterans) who had had negative gender-related experiences in VA, indicated that they were hesitant to seek mental health treatment within VA. Age as it was related to life stage was also a barrier to treatment engagement (initiation and retention). Some Veterans with young children reported that childcare was an extra hurdle to engage in therapy, though most Veterans in our sample were married and reported that they had options for childcare.

#### Enabling factors

Family support for treatment were key enabling factors for treatment engagement. Support partners provided emotional, logistical, and advocacy support to help Veterans get into and attend treatment; support partners described how they navigated the VA system, drove Veterans to appointments, and provided childcare. One Veteran said, “My wife makes sure I get to my appointments. My mother, her job was check on me every time. When she died my brother took on that responsibility. So he texts me every day. And my wife she makes sure I’m okay every day, mentally” (female Veteran). Another Veteran shared about his wife, “I get a lot of encouragement and the best thing of all is that if I ever need something, I have someone that I can confide in, trust, and to assist me with…everything, actually” (Veteran).

Several dyads in the study had participated in marital therapy together. They did not directly link use of marital therapy with use of PTSD therapy but described how helpful marital therapy had been for them to understand PTSD generally. One support partner reported that marriage counseling improved his and the Veteran’s communication around her PTSD management and had helped to recognize her PTSD symptoms,

“We got a good therapist for our marriage counseling and someone that’s more straightforward and that’s what both of us needed. … It’s helped give her that aspect—Hey, okay, this is what’s going on—Kind of identifying where the issue is, rather than just sitting there like—Okay, yeah, I’m mad. But why am I mad—kind of thing.” (Support Partner)

Economic enabling factors did not emerge as an important influencer of treatment engagement in this sample. In fact, participants described how support partners helped them to overcome logistical barriers, such as transportation, that might otherwise be addressed by economic resources.

#### Need factors

The qualitative findings suggest that severity of mental health symptoms was a primary driver of engaging in mental health care. Veterans discussed how they sought care due to a perceived need for treatment because of how PTSD symptoms negatively impacted their lives. In one extreme example, a Veteran who had tried, at his wife’s suggestion, to engage in mental health therapy to help him regain his sense of self after he returned from Afghanistan shared, “If I was going how I was, I’d be dead in a year and a half. Or divorced” (Veteran). On the other hand, PTSD symptoms, such as avoidance, also interfered with engagement in mental health care. Several Veterans cited that their PTSD symptoms lead to a fear of being around other people which prevented them from seeking care in a group setting. One Veteran said, “I don’t care to socialize with others—I mean, even friends. So to do [PTSD therapy] in a group setting with people that I don’t know was out of my comfort zone. It gave me a lot of anxiety” (Veteran). Veterans also described how co-occurring health conditions, such as cancer, drowsiness, depression, and headaches, interfered in their ability to attend treatment. Cognitive conditions, such as traumatic brain injury and substance use, made it difficult for the Veteran to advocate for their treatment needs.

### Multiple methods findings

Table 4 shows how the quantitative findings and themes from the qualitative data were sorted into broad constructs of the Andersen model and then arrayed to build a more complete picture of determinants of treatment engagement. In the quantitative models, predisposing factors, such as gender, age and race/ethnicity, did not influence initiation or retention. Though the qualitative data show gender and age could be important through negative gender-related experiences with the health system and age-related life factors, such as parenting. Positive treatment beliefs emerged as motivators of engagement in the interviews, but treatment belief was not a variable that was available in the quantitative datasets. The quantitative models suggested that not being married drove therapy engagement. We were unable to explore this link directly in the qualitative data because virtually all the Veterans in the sample were married. Yet, there was agreement across both data sources that marital therapy may play a role in promoting PTSD treatment engagement. Economic factors did not appear to be salient in either analysis. The quantitative data showed that having more health needs led to more mental health therapy engagement whereas the qualitative findings suggested that severity of mental health symptoms were the underlying drivers.

**Table 4:** Joint display of results on drivers of the three outcomes (treatment initiation, treatment retention and treatment engagement)

**Theme**	**Quantitative support?**		**Direct qualitative support?**
	*Treatment initiation*	*Treatment retention*	*Treatment engagement*
Predisposing	No	No	Yes Treatment attitudes
Enabling	No	Yes -Not married -Received marital therapy in VA	Yes, Social -Family encouragement and positive social norms around mental health treatment No, Economic
Need	Yes -Higher risk score	Yes -Substance use diagnosis -VA ED visit -Count of PTSD medication refills -Higher risk score	Yes -PTSD symptoms drive initiation; may interfere with retention -Other health conditions inhibit treatment engagement

## Discussion

This study uses a multiple methods approach applied to rich data sources that represent the perspectives of Veterans and their support partners to examine and explain how factors at Veteran and family levels promote Veteran engagement in PTSD therapy. We aim to clarify which of these factors are most important so that future efforts can design targeted interventions at multiple levels to increase uptake of PTSD therapy. We found that the most important factors for Veteran engagement in mental health therapy were Veteran and family treatment beliefs, family support and encouragement for treatment, prior use of marital therapy, and the need for mental health care. In contrast to past quantitative studies [12, 14, 28, 31, 36, 39, 43], but in agreement with others [26], we did not find that predisposing demographic characteristics, including gender, age, race, Latino ethnicity, and education, played a role in treatment engagement. However, as the Nosos risk score includes information about prior utilization, it is possible that this variable captures need as well as other predisposing factors that we can’t separate. Furthermore, the Nosos score reflects greater need for medical services and is not specific to need for PTSD therapy. The fact that Nosos score was more predictive of PTSD service use than mental health diagnoses suggests that perhaps engagement in VA services may be an important driver of PTSD therapy use. However, we are unable to tease this out in the quantitative data. Yet, the findings from the qualitative interviews suggest that the influence of age and gender on treatment engagement may have been related to more distal factors, such as life stage and having young children, which could be one reason that gender and age did not show up in the quantitative models as primary drivers. Treatment beliefs are a key predisposing factor. Interview participants talked about how their beliefs about therapy effectiveness played a role in their engagement in therapy [24, 42], but treatment beliefs are not observable in administrative data.

Social enabling resources were a key component of promoting engagement in treatment. We found strong support for a new potential driver of treatment engagement—marital therapy—which was described by interview participants as relevant for improving interpersonal communication around mental illness. Furthermore, the willingness of both individuals to engage in marital therapy suggests that participants found value in mental health treatment generally and were willing to prioritize mental health service use. The mechanism between family therapy and subsequent treatment engagement for PTSD needs further exploration. Our findings across the two data sources diverged around marital status. The machine learning models identified not being married to be a predictor of therapy retention, but our qualitative findings suggest that family members, primarily spouses, were instrumental in Veterans’ decisions to seek and attend therapy, which is consistent with prior findings about the importance of social support [14, 32, 42]. It is possible that in our quantitative sample of younger Veterans, marriage might be a proxy for having children which our participants identified as a barrier to engagement.

Economic enabling resources was not related to therapy engagement. Our sample comprised Veterans who were eligible for VA services and tend to have fewer economic barriers to care, such as copays or deductibles. Also, all participants had a support partner who may have been available to help offset economic-related logistical challenges by providing transportation and other support.

Our findings about need were consistent with existing evidence [19, 33]; medical need was operationalized several ways in the quantitative models and was a key driver in quantitative outcome models. Furthermore, this finding was reinforced by interview participants who described how mental health symptoms prompted them to seek care. However, this story was complex as participants talked about how mental health symptoms, such as avoidance, might also inhibit therapy utilization [6, 34].

### Clinical implications

Andersen considers enabling resources to be the most amenable to intervention [1]. The findings highlight the importance of Veteran treatment beliefs and social norms around the value of treatment. As such, the role of family—a key social enabling resource—should be considered in future research. One interesting avenue for additional inquiry is how family-involved interventions might operate as a gateway to increasing treatment initiation and retention [35, 48]. There are several pathways through which participating in VA-provided family therapy might improve retention in PTSD therapy, including improving how family members engage with the Veteran around mental health issues and how family members encourage Veterans to seek care [32, 47], reducing family conflict, or introducing family members to mental health services at VA.

### Research implications

We demonstrate the need to use multiple types of data when examining complex care-seeking behavior. For example, the machine learning model that predicted initiation of therapy was less accurate than the model predicting therapy retention. This reflects findings from another study that examined predictors of initiation of and retention in evidenced-based PTSD therapies using medical record data from 427 Veterans and found no predictors of treatment initiation [25]. One reason for our finding could be that organizational and provider factors [35, 38, 51] may be more important for promoting access to treatment than individual and family factors. It is possible that our therapy retention models were better able to classify individuals correctly because once Veterans have started treatment, health needs and social support become more important for maintaining treatment engagement [20, 42]; our models were able to account for these factors through combined survey and administrative data. Future research in this area is needed given the exploratory nature of machine learning models. However, these efforts should carefully consider the different mechanisms that underlie the outcomes of interest and whether the data source and variables available are adequate to capture the complex drivers of mental health engagement, especially in light of the limitations of administrative data.

### Strengths and limitations

This study has some limitations. First, the quantitative and qualitative data defined treatment engagement slightly differently; we focused on initiation of and retention in a new episode of mental health therapy in the quantitative models and in the interviews, we talked generally about use of mental health care and were not always able to distinguish what influenced initiation versus retention in the qualitative data. Second, respondents in our samples had applied to the VA PCAFC program and therefore they might be different than other Veterans/support partners who had not applied and this may limit the generalizability of our findings beyond Iraq/Afghanistan Veterans in PCAFC. However, currently in the VA system, program data from the Caregiver Support Program is the only way to identify Veterans who have a support partner and so this was a necessary approach to identify support partners. Because of the lack of systematic identification of support partners, there were no other studies that have compared Afghanistan and Iraq Veterans who enrolled in PCAFC to those who did not, but studies have compared differences among this group who enrolled in the program versus Veterans who applied but were denied. One study of Veterans with PTSD who enrolled in PCAFC were more likely than Veterans who were denied to be white, have a traumatic brain injury (TBI), have a higher rating for military service connected disability, and be a spouse (CITATION BLINDED FOR REVIEW). As a result, we expect that our results might generalize to Veterans with PTSD who have a comorbid TBI, are white, have a high level of service connection, and are married to their support partner. We might also expect that the importance of “need for services” represented by the Nosos risk score on treatment initiation might be confounded with enrollment in PCAFC as PCAFC is associated with increases in primary care, mental health care, and specialty care service utilization (CITATAION BLINDED FOR REVIEW). Third, while the qualitative and quantitative samples included individuals who had applied to the VA PCAFC, all individuals in the quantitative sample had engaged in PCAFC services for at least 3 months and may have received additional VA resources or were different in some way than other Veterans, including Veterans in the qualitative sample, who had not all been approved for PCAFC. These sample differences could have limited comparability of the quantitative and qualitative results. Fourth, while we defined “adequate dose” as 8+ sessions, some Veterans do achieve clinical benefit with fewer sessions, and so this might have inhibited our ability to accurately predict therapy completion. Last, interview participants had prior experiences with mental health therapy, so the perspectives presented therein may differ from those of individuals who have no treatment experience. Therefore, the findings about treatment attitudes may reflect satisfaction with treatment as opposed to a true pre-disposing factor of the past treatment episodes discussed during the interviews.

However, our results are novel because they uncover the most important factors of therapy engagement by using a comprehensive approach that considers factors at multiple levels and using multiple types of data that represent several perspectives. In comparison, past literature has generally applied had a narrower scope and may have been unable to assess such a broad range of factors and viewpoints. The strengths of this study include our use of quantitative and qualitative data to provide a more comprehensive understanding of the complex process of PTSD treatment engagement. This study is also among the few that we know of to consider the perspectives of support partners [32, 46].

## Conclusions

Our multiple methods and multiple perspectives study suggests that Veteran pre-disposing and need factors and family enabling factors influence treatment engagement, but that drivers of starting treatment and adhering to treatment are not the same. Administrative data studies may not adequately capture important drivers of mental health engagement, including treatment attitudes, social support, and some organizational barriers. A key finding was the role of support partners as a potential avenue for increasing treatment engagement. Future efforts to identify different ways to involve support partners within the care process is a promising avenue to increase PTSD therapy uptake. While our study offers a comprehensive and integrated understanding of what and how certain factors influence engagement in PTSD therapy, these findings need to be validated in other samples.

## Supplementary Information

**Additional file 1.** Methodological Appendix. Given the complexity of a mixed methods project that includes a secondary analysis using machine learning and qualitative interviews, we included the technical details of the machine learning approach in the methodological appendix.

**Additional file 2.**

**Additional file 3.** Script: Family member of Veteran in treatment. This script was administered to family members of Veterans who were interviewed prior to starting mental health therapy for PTSD.

**Additional file 4.** Script: Family member of Veteran prior to treatment. This script was administered to family members of Veterans who were interviewed while they were in therapy for PTSD.

**Additional file 5.** Script: Veteran in treatment. This script was administered to Veterans who were interviewed while they were in therapy for PTSD.

**Additional file 6.** Script: Veteran prior to treatment. This script was administered to Veterans who were interviewed prior to starting mental health therapy for PTSD.

## Data Availability

Data from Veteran medical records and claims are not available, but requests for the qualitative data and statistical code can be sent to Megan Shepherd-Banigan, megan-shepherd-banigan@va.gov.

## Abbreviations

AUC: Area Under the ROC Curve
CES-D: Center for Epidemiological Studies-Depression
EHR: Electronic Health Record
ICD-9: International Classification of Disease, 9^th^ Revision
PCAFC: VA Program for Comprehensive Assistance for Family Caregivers
PTSD: Posttraumatic stress disorder
VA: Veterans Affairs

## References

[1] Andersen RM (1995). Revisiting the behavioral model and access to medical care: does it matter?. J Health Soc Behav.

[2] Andresen EM, Malmgren JA, Carter WB, Patrick DL (1994). Screening for depression in well older adults: evaluation of a short form of the CES-D (Center for Epidemiologic Studies Depression Scale). Am J Prev Med.

[3] Bedard M, Molloy DW, Squire L, Dubois S, Lever JA, O’Donnell M (2001). The Zarit Burden Interview: a new short version and screening version. Gerontologist.

[4] Bradley EH, McGraw SA, Curry L, Buckser A, King KL, Kasl SV, Andersen R (2002). Expanding the Andersen model: the role of psychosocial factors in long-term care use. Health Serv Res.

[5] Breiman L (2001). Random forests. Mach Learn.

[6] Bryant RA, Moulds ML, Mastrodomenico J, Hopwood S, Felmingham K, Nixon RDV (2007). Who drops out of treatment for post-traumatic stress disorder?. Clin Psychol.

[7] Collins KMT, Onwuegbuzie AJ, Jiao QG (2016). A mixed methods investigation of mixed methods sampling designs in social and health science research. J Mixed Methods Res.

[8] Department of Veteran Affairs. Management of post-traumatic stress disorder and acute stress reaction. Retrieved from Washington, DC; 2010. https://www.healthquality.va.gov/guidelines/MH/ptsd/.

[9] Doran JM, Pietrzak RH, Hoff R, Harpaz-Rotem I (2017). Psychotherapy utilization and retention in a national sample of veterans with PTSD. J Clin Psychol.

[10] Erbes CR, Kaler ME, Schult T, Polusny MA, Arbisi PA (2011). Mental health diagnosis and occupational functioning in National Guard/Reserve veterans returning from Iraq. J Rehabil Res Dev.

[11] Fulton JJ, Calhoun PS, Wagner HR, Schry AR, Hair LP, Feeling N (2015). The prevalence of posttraumatic stress disorder in Operation Enduring Freedom/Operation Iraqi Freedom (OEF/OIF) Veterans: a meta-analysis. J Anxiety Disord.

[12] Garcia HA, Kelley LP, Rentz TO, Lee S (2011). Pretreatment predictors of dropout from cognitive behavioral therapy for PTSD in Iraq and Afghanistan war veterans. Psychol Serv.

[13] Given CW, Given B, Stommel M, Collins C, King S, Franklin S (1992). The caregiver reaction assessment (CRA) for caregivers to persons with chronic physical and mental impairments. Res Nurs Health.

[14] Gros DF, Price M, Yuen EK, Acierno R (2013). Predictors of completion of exposure therapy in OEF/OIF veterans with posttraumatic stress disorder. Depress Anxiety.

[15] Haller M, Myers US, McKnight A, Angkaw AC, Norman SB (2016). Predicting engagement in psychotherapy, pharmacotherapy, or both psychotherapy and pharmacotherapy among returning veterans seeking PTSD treatment. Psychol Serv.

[16] Hamilton AB. Qualitative methods in rapid turn-around health services research. In: Paper presented at the Health Services Research & Development: spotlight on women’s health. 2013.

[17] Hmelo-Silver CE (2003). Analyzing collaborative knowledge construction. Comput Educ.

[18] Hoge CW, Grossman SH, Auchterlonie JL, Riviere LA, Milliken CS, Wilk JE (2014). PTSD treatment for soldiers after combat deployment: low utilization of mental health care and reasons for dropout. Psychiatr Serv.

[19] Hundt NE, Barrera TL, Mott JM, Mignogna J, Yu HJ, Sansgiry S (2014). Predisposing, enabling, and need factors as predictors of low and high psychotherapy utilization in veterans. Psychol Serv.

[20] Hundt NE, Robinson A, Arney J, Stanley MA, Cully JA (2015). Veterans’ Perspectives on benefits and drawbacks of peer support for posttraumatic stress disorder. Mil Med.

[21] Ishwaran H, Kogalu UB. Package ‘randomForestSRC’ (version 2.9.2): CRAN. 2019.

[22] Johnson RB, Onwuegbuzie AJ, Turner LA (2016). Toward a definition of mixed methods research. J Mixed Methods Res.

[23] Jones D, Kazis L, Lee A, Rogers W, Skinner K, Cassar L (2001). Health status assessments using the Veterans SF-12 and SF-36: methods for evaluating otucomes in the Veterans Health Administration. J Ambul Care Manage.

[24] Kehle-Forbes SM, Gerould H, Polusny MA, Sayer NA, Partin MR (2020). “It leaves me very skeptical” messaging in marketing prolonged exposure and cognitive processing therapy to veterans with PTSD. Psychol Trauma.

[25] Kehle-Forbes SM, Meis LA, Spoont MR, Polusny MA (2016). Treatment initiation and dropout from prolonged exposure and cognitive processing therapy in a VA outpatient clinic. Psychol Trauma.

[26] Keller SM, Tuerk PW (2016). Evidence-based psychotherapy (EBP) non-initiation among veterans offered an EBP for posttraumatic stress disorder. Psychol Serv.

[27] Liaw A, Weiner M. Package ‘random forest’ (version 4.6): CRAN. 2018.

[28] Lu MW, Duckart JP, O'Malley JP, Dobscha SK (2011). Correlates of utilization of PTSD specialty treatment among recently diagnosed veterans at the VA. Psychiatr Serv.

[29] Ma JE, Grubber J, Coffman CJ, Wang V, Hastings SN, Allen KD (2022). Identifying family and unpaid caregivers in electronic health records: descriptive analysis. JMIR Form Res.

[30] Maguen S, Li Y, Madden E, Seal KH, Neylan TC, Patterson OV (2019). Factors associated with completing evidence-based psychotherapy for PTSD among veterans in a national healthcare system. Psychiatry Res.

[31] Maguen S, Madden E, Neylan TC, Cohen BE, Bertenthal D, Seal KH (2014). Timing of mental health treatment and PTSD Symptom improvement among Iraq and Afghanistan veterans. Psychiatr Serv.

[32] Meis LA, Noorbaloochi S, Hagel Campbell EM, Erbes CR, Polusny MA, Velasquez TL (2019). Sticking it out in trauma-focused treatment for PTSD: it takes a village. J Consult Clin Psychol.

[33] Mott JM, Mondragon S, Hundt NE, Beason-Smith M, Grady RH, Teng EJ (2014). Characteristics of U.S. veterans who begin and complete prolonged exposure and cognitive processing therapy for PTSD. J Trauma Stress.

[34] Ouimette P, Vogt D, Wade M, Tirone V, Greenbaum MA, Kimerling R (2011). Perceived barriers to care among veterans health administration patients with posttraumatic stress disorder. Psychol Serv.

[35] Rosen CS, Matthieu MM, WiltseyStirman S, Cook JM, Landes S, Bernardy NC (2016). A Review of studies on the system-wide implementation of evidence-based psychotherapies for posttraumatic stress disorder in the Veterans Health Administration. Adm Policy Ment Health.

[36] Sayer NA, Clothier B, Spoont M, Nelson DB (2007). Use of mental health treatment among veterans filing claims for posttraumatic stress disorder. J Trauma Stress.

[37] Sayer NA, Noorbaloochi S, Frazier P, Carlson K, Gravely A, Murdoch M (2010). Reintegration problems and treatment interests among Iraq and Afghanistan combat veterans receiving VA medical care. Psychiatr Serv.

[38] Sayer NA, Wiltsey-Stirman S, Rosen CS, Bernardy NC, Spoont MR, Kehle-Forbes SM (2021). Investigation of therapist effects on patient engagement in evidence-based psychotherapies for posttraumatic stress disorder in the Veterans Health Administration. J Trauma Stress.

[39] Seal KH, Maguen S, Cohen B, Gima KS, Metzler TJ, Ren L (2010). VA mental health services utilization in Iraq and Afghanistan veterans in the first year of receiving new mental health diagnoses. J Trauma Stress.

[40] Siriseriwan W. Package ‘smotefamily’ (version 1.3.1): CRAN. 2019.

[41] Spoont MR, Murdoch M, Hodges J, Nugent S (2010). Treatment receipt by veterans after a PTSD diagnosis in PTSD, mental health, or general medical clinics. Psychiatr Serv.

[42] Spoont MR, Nelson DB, Murdoch M, Rector T, Sayer NA, Nugent S, Westermeyer J (2014). Impact of treatment beliefs and social network encouragement on initiation of care by VA service users with PTSD. Psychiatr Serv.

[43] Spoont MR, Nelson DB, Murdoch M, Sayer NA, Nugent S, Rector T, Westermeyer J (2015). Are there racial/ethnic disparities in VA PTSD treatment retention?. Depress Anxiety.

[44] Tarlow BJ, Wisniewski SR, Belle SH, Rubert M, Ory MG, Gallagher-Thompson D (2016). Positive aspects of caregiving. Res Aging.

[45] Thomas E, Magilvy JK (2011). Qualitative rigor or research validity in qualitative research. J Spec Pediatr Nurs.

[46] Thompson-Hollands J, Burmeister LB, Rosen CS, O'Dougherty M, Erickson EPG, Meis LA (2019). Veterans with poor PTSD treatment adherence: exploring their loved ones’ experience of PTSD and understanding of PTSD treatment. Psychol Serv.

[47] Thompson-Hollands J, Burmeister LB, Rosen CS, O'Dougherty M, Erickson EPG, Meis LA (2021). Veterans with poor PTSD treatment adherence: exploring their loved ones’ experience of PTSD and understanding of PTSD treatment. Psychol Serv.

[48] Thompson-Hollands J, Strage M, DeVoe ER, Beidas RS, Sloan DM (2020). Development of a brief adjunctive intervention for family members of veterans in individual PTSD treatment. Cogn Behav Pract.

[49] Van Houtven CH, Smith VA, Stechuchak KM, Shepherd-Banigan M, Hastings SN, Maciejewski ML, Wieland GD, Olsen MK, Miller KEM, Kabat M, Henius J, Campbell-Kotler M, Oddone EZ. Comprehensive Support for Family Caregivers: Impact on Veteran Health Care Utilization and Costs. Med Care Res Rev. 2019;76(1):89-114. 10.1177/1077558717697015. Epub 2017 Apr 1. PMID: 29148338; PMCID: PMC5726944. 10.1177/1077558717697015 PMC5726944 29148338

[50] Wagner TH, Upadhyay A, Cowgill E, Stefos T, Moran E, Asch SM, Almenoff P (2016). Risk adjustment tools for learning health systems: a comparison of DxCG and CMS-HCC V21. Health Serv Res.

[51] Zubkoff L, Carpenter-Song E, Shiner B, Ronconi JM, Watts BV (2016). Clinicians’ perception of patient readiness for treatment: an emerging theme in implementation science?. Adm Policy Ment Health.

