# Supplementary material for: Exploring the importance of predisposing, enabling, and need factors for promoting Veteran engagement in mental health therapy for post-traumatic stress: a multiple methods study
Source: BMC Psychiatry. 2023 May 27;23:372. doi: 10.1186/s12888-023-04840-7 (PMC10219808; doi:10.1186/s12888-023-04840-7)
Supplement: Supplementary file 1 — Additional file 1. Methodological Appendix. Given the complexity of a mixed methods project that includes a secondary analysis using machine learning and qualitative interviews, we included the technical details of the machine learning approach in the methodological appendix. [file 12888_2023_4840_MOESM1_ESM.pdf]

Methodological Appendix

1.1. Survey sample. The survey sample included 1,407 caregivers who responded to a web or telephone based survey. To recruit participants, invitations were emailed to 10,000 caregivers of Veterans who had applied to PCAFC. A subset of those caregivers (n=8,118) comprised a 10-percent stratified sample of caregivers applying to PCAFC from each Veteran Affairs Medical Center (VAMC) and a national sample of 1,882 caregivers who were surveyed 9 months earlier and were re-contacted. Participants were informed of the study purpose and benefits/risks of the study. Starting the survey implied informed consent. Data was collected between September and October 2015. The response rate was 14% (REFERENCE BLINDED FOR REVIEW).

2.1 Full Outcome Definitions. To define the mental health treatment outcomes, we used Current Procedural Terminology (CPT) codes that denote Individual or Group Behavioral Counseling, had an associated PTSD ICD-10 diagnosis (F43.10, F43.11, F43.13), and provider classifications indicating the provision of mental health services (e.g., "Clinical Psychologist", "Marriage & Family Therapy") (Spoont, personal communication 12/6/19). CPT codes that denote telephone-based therapy were excluded as those codes are generally not used to code therapy services (Frayne et al., 2018). Specifically, we used the list of CPT codes designated by Maguen and colleagues (Maguen et al., 2018) which included all mental health services stop codes (500-599) and stop codes 125, 156, 157, and 292. If a Veteran had more than one qualifying visit on the same day, we counted those visits as one single visit. Initiation of a treatment episode was defined as at least two sessions of therapy received on different days, but that occurred within 21 days of one another between December 1, 2015 and September 30, 2017. We designated two visits to maximize the possibility that Veterans engaged in therapy because the first visit could indicate an evaluation visit and not actual therapy and we limited the space between treatment to

21 days as visits that occur 30 days apart might indicate case management and not therapy (Spoont, personal communication 12/6/19). Completion of an adequate dose of treatment (referred to as "adherence") was defined using a previously accepted definition (Spoont et al., 2010) as the receipt of at least 8 sessions of therapy received within 180 days between December 1, 2015 and September 30, 2017.

3.1 Rationale for Machine Learning. We used a random forest algorithm for binary outcomes to search for patterns in the data about the importance of a range of Veteran and support partner-level predisposing, enabling, and need variables on Veteran use of VA-provided mental health therapy to identify new insights and develop hypotheses. When used in accordance with accepted procedures (James et al., 2017) (i.e., training/test datasets, parameter tuning, etc.), some machine learning approaches, such as random forests, are a rigorous approach to explore data and are not constrained by limitations of regression methods, such as inflated Type I errors and mis-specified outcome distributions (Breiman, 2001).

The random forest algorithm for binary outcomes is based on a classification decision-tree framework. Decision trees repeatedly split the sample into homogenous subgroups based on groups of characteristics that predict the same outcome class. Random forests then use randomly drawn samples to construct many trees, average the results across all trees, and rank the relative importance of each driver based on that factor's influence on the outcome across all samples. Random forests do not produce estimates of effect or assign a direction of effect, only the relative importance of a single variable compared with the other variables included in the model.

4.1 Detailed Statistical Analysis Approach. We created a separate dataset for each outcome. Missing data were imputed for each outcome cohort using adaptive tree imputations—a random forest approach that iteratively and randomly imputes missing data using randomly drawn non-

missing in-sample data (RStudio package: *randomForestSRC version 2.9.3*) that returns a complete dataset. Then, we split the sample into each dataset into a 70% training dataset and 30% testing dataset; this split follows conventional approaches. We then rebalanced the dataset for the adherence outcome. Approximately 25% of the sample was adherent; this 25%:75% split in the outcome across the sample is not considered to be sufficiently balanced and could bias the decision trees thereby biasing the average generated by the random forest algorithm. Therefore, we applied a sample rebalancing technique (RStudio package: *smotefamily version 1.3.1*) (Siriseriwan, 2019) to simulate data for cases who completed an adequate dose so that the outcome was balanced.

For each outcome dataset, we tuned the parameters in the training dataset to identify the optimal number of trees and the number of variables that are randomly sampled at each split in the tree, and ran the best model in the test dataset to generate the area under the curve (AUC) metric to assess how predictive the model was (AUC ranges from 0 to 1 and an AUC of 0.50 indicates that the model correctly classifies observations at the same rate as chance).

We then ran the best model in the full dataset to estimate the relative importance of each variable in driving the outcome using the RStudio package *randomForest* version 4.6-14 (Breiman et al., 2018). Random forests were estimated using 1,000 bootstrapped trees. As a robustness check for the main models, we partitioned the dataset into 5 random subsamples and ran the random forest algorithm to assess whether results were consistent across these subsamples.

To further protect against overfitting and the exploratory nature of this type of analysis, we examined the most influential variables across several other random forests algorithms. For both the initiation and adherence outcomes, we ran a different random forest algorithm using the RStudio *randomForestSRC version 2.9.3* package (Ishwaran et al., 2019). Additionally, for the

adherence outcome only we ran the random forest algorithm from the *randomForest* version 4.6-14 in the non-balanced sample. We did not do this for the initiation outcome because we did not need to rebalance the sample as the outcome was evenly split among the participants. Two analysts (MSB, VAS) compared the most influential variables across all of these models by outcome and selected the variables that appeared in all models. We then estimated the bi-variate association using regression models to understand the direction of effect; we do not present the coefficients or inferences as we conducted the statistical test using the same data to select the variables. All analyses were done in RStudio version 4.0.2 and SAS version 9.4.

References

Breiman, L. (2001). Random Forests. *Machine Learning, 45*(1), 5-32. doi:10.1023/a:1010933404324

Breiman, L., Cutler, A., Liaw, A., & Wiener, M. (2018). *Breiman and Cutler's Random Forests for Classification and Regression*. Retrieved from https://cran.r-project.org/web/packages/randomForest/randomForest.pdf

Frayne, S., Phibbs, C., Saechao, F., Friedman, S. A., Shaw, J., Romodan, Y., . . . Haskell, S. (2018). *Sourcebook: Women Veterans in the Veterans Health Administration*. Retrieved from Washington, DC:

Ishwaran, H., & Kogalu, U. B. (2019). Package 'randomForestSRC' (Version 2.9.2): CRAN.

James, G., Witten, D., Hastie, T., & Tibshirani, R. (2017). *An Introduction to Statistical Learning* (8th Ed.). New York, NY: Springer.

Maguen, S., Madden, E., Patterson, O. V., DuVall, S. L., Goldstein, L. A., Burkman, K., & Shiner, B. (2018). Measuring Use of Evidence Based Psychotherapy for Posttraumatic Stress Disorder in a Large National Healthcare System. *Administration and Policy in Mental Health, 45*(4), 519-529. doi:10.1007/s10488-018-0850-5

Siriseriwan, W. (2019). Package 'smotefamily' (Version 1.3.1): CRAN.

Spoont, M. R., Murdoch, M., Hodges, J., & Nugent, S. (2010). Treatment receipt by veterans after a PTSD diagnosis in PTSD, mental health, or general medical clinics. *Psychiatric Services, 61*(1), 58-63. doi:10.1176/appi.ps.61.1.58
