# Supplementary material for: Exploring the importance of predisposing, enabling, and need factors for promoting Veteran engagement in mental health therapy for post-traumatic stress: a multiple methods study
Source: BMC Psychiatry. 2023 May 27;23:372. doi: 10.1186/s12888-023-04840-7 (PMC10219808; doi:10.1186/s12888-023-04840-7)
Supplement: Supplementary file 2 — Additional file 2. [file 12888_2023_4840_MOESM2_ESM.pdf]

**Veteran and family member baseline characteristics**		**Treatment initiation**		**Completion of an adequate dose**	
	Total Sample	Yes	No	Yes	No
	n=1237	n=506	n=731	n=312	n=925
**Pre-disposing Factors**					
Veteran gender [b]					
Male, n (%)	1156 (93.5)	464 (40.1)	692 (59.9)	280 (24.2)	876 (75.8)
Female, n (%)	81 (6.5)	42 (51.9)	39 (48.1)	32 (39.5)	49 (60.5)
Family member gender [a, e]					
Male, n (%)	67 (5.4)	30 (44.8)	37 (55.2)	21 (31.3)	46 (68.7)
Female, n (%)	1168 (94.6)	475 (40.7)	693 (59.3)	290 (24.8)	878 (75.2)
Veteran age [b], mean (SD)	40.9 (9.4)	40.4 (9.0)	41.3 (9.7)	40.7 (9.1)	41.0 (9.5)
Family member age [a, e], mean (SD)	42.8 (11.9)	42.7 (12.1)	42.9 (11.8)	43.2 (12.2)	42.7 (11.8)
Veteran race [b, e]					
White, n (%)	794 (65.3)	327 (41.2)	467 (58.8)	202 (25.4)	592 (74.6)
Black, n (%)	236 (19.4)	89 (37.7)	147 (62.3)	53 (22.5)	183 (77.5)
Other [f], n (%)	186 (15.3)	80 (43.0)	106 (57.0)	49 (26.3)	137 (73.7)
Veteran Hispanic/Latino(a) ethnicity [b, e]					
Yes, n (%)	294 (23.9)	124 (42.2)	170 (57.8)	76 (25.9)	218 (74.1)
No, n (%)	936 (76.1)	380 (40.6)	556 (59.4)	235 (25.1)	701 (74.9)
Family member race [a, e]					
White, n (%)	744 (64.6)	294 (39.5)	450 (60.5)	186 (25.0)	558 (75.0)
Black, n (%)	182 (15.8)	71 (39.0)	111 (61.0)	45 (24.7)	137 (75.3)

Other [f], n (%)	225 (19.5)	102 (45.3)	123 (54.7)	56 (24.9)	169 (75.1)
Family member Hispanic/Latino ethnicity(a) [a, e]					
Yes, n (%)	294 (24.3)	130 (44.2)	164 (55.8)	76 (25.9)	218 (74.1)
No, n (%)	917 (75.7)	363 (39.6)	554 (60.4)	226 (24.6)	691 (75.4)
No. of people living in the household [a, e], mean (SD)	3.5 (1.4)	3.4 (1.3)	3.6 (1.5)	3.5 (1.3)	3.5 (1.4)
No. of persons under age 18 living in the family member's household [a, e], mean (SD)	1.2 (1.2)	1.1 (1.1)	1.2 (1.3)	1.0 (1.1)	1.2 (1.3)
Family member highest level of education [a, e]					
High school graduate or less, n (%)	253 (21.0)	98 (38.7)	155 (61.3)	55 (21.7)	198 (78.3)
Some college, Associate's degree, trade school, n (%)	630 (52.2)	259 (41.1)	371 (58.9)	162 (25.7)	468 (74.3)
Bachelor's degree or higher, n (%)	323 (26.8)	131 (40.6)	192 (59.4)	86 (26.6)	237 (73.4)
**Enabling Factors**					
Veteran and family member partnered [a, e]					
Spouses/significant other, n (%)	1061 (86.1)	428 (40.3)	633 (59.7)	258 (24.3)	803 (75.7)
Not spouse/significant other, n (%)	172 (13.9)	77 (44.8)	95 (55.2)	53 (30.8)	119 (69.2)
Veteran marital status [b, e]					
Married, n (%)	976 (79.1)	388 (39.8)	588 (60.2)	234 (24.0)	742 (76.0)
Not married, n (%)	258 (20.9)	117 (45.3)	141 (54.7)	77 (29.8)	181 (70.2)
Family member marital status [a, e]					
Married, n (%)	1059 (86.8)	425 (40.1)	634 (59.9)	258 (24.4)	801 (75.6)
Not married, n (%)	161 (13.2)	72 (44.7)	89 (55.3)	49 (30.4)	112 (69.6)
Family member reported Veteran financial situation [a, e]					

He/she is in good shape. He/she is able to save and plan for the future., n (%)	67 (6.4)	18 (26.9)	49 (73.1)	9 (13.4)	58 (86.6)
He/she is okay. He/she is saving a little. He/she is able to provide for his/her needs., n (%)	448 (42.6)	173 (38.6)	275 (61.4)	109 (24.3)	339 (75.7)
He/she is barely getting by. He/she has to budget carefully and is not able to plan for the future., n (%)	397 (37.8)	171 (43.1)	226 (56.9)	103 (25.9)	294 (74.1)
He/she is falling behind. He/she has to use savings or go further into debt to pay bills., n (%)	100 (9.5)	48 (48.0)	52 (52.0)	29 (29.0)	71 (71.0)
He/she is in serious financial trouble, and can't quite see how he/she is going to make it., n (%)	39 (3.7)	18 (46.2)	21 (53.8)	12 (30.8)	27 (69.2)
Family member economic situation [a, e]					
I am in good shape. I am able to save and plan for the future., n (%)	56 (5.2)	22 (39.3)	34 (60.7)	14 (25.0)	42 (75.0)
I am okay. I am saving a little, and I am able to provide for my needs., n (%)	468 (43.3)	173 (37.0)	295 (63.0)	114 (24.4)	354 (75.6)
I am barely getting by. I have to budget carefully, and I am not able to plan for the future., n (%)	433 (40.1)	188 (43.4)	245 (56.6)	109 (25.2)	324 (74.8)
I am falling behind. I have to use savings or go further into debt to pay my bills, n (%).	102 (9.4)	45 (44.1)	57 (55.9)	30 (29.4)	72 (70.6)
I am in serious financial trouble, and can't quite see how I am going to make it., n (%)	21 (1.9)	10 (47.6)	11 (52.4)	5 (23.8)	16 (76.2)
Family member Perceived Financial Strain score [a, e], mean (SD)	9.0 (3.3)	9.1 (3.2)	8.9 (3.4)	9.3 (3.1)	8.9 (3.4)
No. of years Veteran enrolled in VA care [c], mean (SD)	7.1 (3.9)	7.1 (4.0)	7.1 (3.9)	7.2 (4.0)	7.1 (3.9)
Family member employment status [a, e]					
Working for paid employment, n (%)	444 (42.1)	174 (39.2)	270 (60.8)	108 (24.3)	336 (75.7)
Not working for paid employment, n (%)	611 (57.9)	254 (41.6)	357 (58.4)	155 (25.4)	456 (74.6)

Average no. of days in the week that family member provides for Veteran [a, e], mean (SD)	6.8 (0.6)	6.8 (0.6)	6.8 (0.6)	6.8 (0.6)	6.8 (0.7)
Family member health status [a, e]					
Excellent, n (%)	70 (6.6)	23 (32.9)	47 (67.1)	15 (21.4)	55 (78.6)
Very good, n (%)	263 (24.8)	110 (41.8)	153 (58.2)	63 (24.0)	200 (76.0)
Good, n (%)	469 (44.2)	191 (40.7)	278 (59.3)	122 (26.0)	347 (74.0)
Fair, n (%)	234 (22.1)	95 (40.6)	139 (59.4)	57 (24.4)	177 (75.6)
Poor, n (%)	24 (2.3)	11 (45.8)	13 (54.2)	6 (25.0)	18 (75.0)
Family member subjective burden score (Zarit Burden) [a, e], mean (SD)	15.4 (10.0)	16.0 (9.8)	15.0 (10.1)	16.0 (9.6)	15.2 (10.1)
Family member depressive symptoms score (CESD-10) [a, e], mean (SD)	8.5 (5.9)	9.0 (5.9)	8.1 (5.8)	9.2 (5.8)	8.2 (5.8)
Family member Positive aspects of caregiving score [a, e], mean (SD)	34.9 (8.3)	34.3 (8.5)	35.3 (8.2)	34.0 (8.7)	35.2 (8.2)
Family member missed scheduled doctor's appointments in past year [a, e]					
Yes, n (%)	227 (22.0)	85 (37.4)	142 (62.6)	47 (20.7)	180 (79.3)
No, n (%)	803 (78.0)	335 (41.7)	468 (58.3)	210 (26.2)	593 (73.8)
Family member had flu shot in past year [a, e]					
Yes, n (%)	366 (35.7)	153 (41.8)	213 (58.2)	95 (26.0)	271 (74.0)
No, n (%)	659 (64.3)	267 (40.5)	392 (59.5)	163 (24.7)	496 (75.3)
Veteran drive time to closest VA primary care facility in minutes [c, e], mean (SD)	20.9 (14.2)	20.8 (14.6)	21.0 (14.0)	20.2 (14.3)	21.1 (14.2)
Veteran drive time to closest VA secondary care facility in minutes [c, e], mean (SD)	50.0 (38.1)	47.2 (38.1)	51.8 (38.1)	45.8 (34.6)	51.4 (39.2)

Veteran drive time to closest VA tertiary care facility in minutes [c, e], mean (SD)	95.7 (71.5)	92.9 (73.0)	97.7 (70.5)	88.5 (67.7)	98.2 (72.6)
Average no. of days the Veteran has been enrolled in the PCAFC [d, e], mean (SD)	602.9 (418.9)	578.9 (400.1)	619.4 (430.9)	603.2 (408.2)	602.7 (422.6)
Family Member used the Caregiver Support Line services [a, e]					
Yes, n (%)	250 (25.0)	88 (35.2)	162 (64.8)	52 (20.8)	198 (79.2)
No, n (%)	750 (75.0)	315 (42.0)	435 (58.0)	197 (26.3)	553 (73.7)
CSP has helped caregiver to assist the Veteran in becoming more engaged in health care [a, e]					
Strongly disagree, n (%)	31 (3.0)	10 (32.3)	21 (67.7)	3 (9.7)	28 (90.3)
Disagree, n (%)	37 (3.5)	11 (29.7)	26 (70.3)	5 (13.5)	32 (86.5)
Neither agree or disagree, n (%)	196 (18.7)	81 (41.3)	115 (58.7)	52 (26.5)	144 (73.5)
Agree, n (%)	401 (38.3)	172 (42.9)	229 (57.1)	111 (27.7)	290 (72.3)
Strongly agree, n (%)	381 (36.4)	155 (40.7)	226 (59.3)	92 (24.1)	289 (75.9)
CSP has helped family member to support progress in my Veteran's rehabilitation or treatment [a, e]					
Strongly disagree, n (%)	37 (3.5)	13 (35.1)	24 (64.9)	6 (16.2)	31 (83.8)
Disagree, n (%)	40 (3.8)	15 (37.5)	25 (62.5)	10 (25.0)	30 (75.0)
Neither agree or disagree, n (%)	191 (18.3)	82 (42.9)	109 (57.1)	54 (28.3)	137 (71.7)
Agree, n (%)	372 (35.6)	147 (39.5)	225 (60.5)	85 (22.8)	287 (77.2)
Strongly agree, n (%)	406 (38.8)	172 (42.4)	234 (57.6)	109 (26.8)	297 (73.2)
**Need Factors**					
Veteran service connection (SC) status [c, e]					
Not SC or SC <70%, n (%)	119 (9.6)	52 (43.7)	67 (56.3)	27 (22.7)	92 (77.3)

SC 70% - less than 100%, n (%)	563 (45.6)	226 (40.1)	337 (59.9)	136 (24.2)	427 (75.8)
SC 100%, n (%)	554 (44.8)	227 (41.0)	327 (59.0)	148 (26.7)	406 (73.3)
No. of years since Veteran's first PTSD diagnosis in VA [c], mean (SD)	5.3 (3.1)	5.3 (3.1)	5.3 (3.1)	5.3 (3.1)	5.2 (3.1)
No. of Veteran outpatient prescription PTSD medication refills in past 12 months [c], mean (SD)	1.8 (2.8)	1.9 (2.9)	1.7 (2.7)	2.1 (3.0)	1.7 (2.7)
Compared to one year ago, how would you rate his/her emotional problems (such as feeling anxious, depressed or irritable) now? [a, e]					
Much better, n (%)	27 (2.8)	7 (25.9)	20 (74.1)	6 (22.2)	21 (77.8)
Slightly better, n (%)	143 (14.6)	52 (36.4)	91 (63.6)	31 (21.7)	112 (78.3)
About the same, n (%)	412 (42.1)	161 (39.1)	251 (60.9)	105 (25.5)	307 (74.5)
Slightly worse, n (%)	242 (24.7)	104 (43.0)	138 (57.0)	57 (23.6)	185 (76.4)
Much worse, n (%)	155 (15.8)	80 (51.6)	75 (48.4)	51 (32.9)	104 (67.1)
Veteran Concurrent Nosos score FY2015 [c, g], mean (SD)	1.5 (1.5)	1.8 (1.7)	1.3 (1.2)	1.8 (1.8)	1.4 (1.3)
Veteran diagnoses assessed in past 12 months					
Depressive disorder diagnosis [c]					
Yes, n (%)	791 (63.9)	359 (45.4)	432 (54.6)	215 (27.2)	576 (72.8)
No, n (%)	446 (36.1)	147 (33.0)	299 (67.0)	97 (21.7)	349 (78.3)
Anxiety disorder diagnosis (not PTSD) [c]					
Yes, n (%)	350 (28.3)	152 (43.4)	198 (56.6)	94 (26.9)	256 (73.1)
No, n (%)	887 (71.7)	354 (39.9)	533 (60.1)	218 (24.6)	669 (75.4)
Bipolar disorder diagnosis [c]					
Yes, n (%)	83 (6.7)	39 (47.0)	44 (53.0)	25 (30.1)	58 (69.9)

No, n (%)	1154 (93.3)	467 (40.5)	687 (59.5)	287 (24.9)	867 (75.1)
Psychotic disorder diagnosis [c]					
Yes, n (%)	65 (5.3)	29 (44.6)	36 (55.4)	15 (23.1)	50 (76.9)
No, n (%)	1172 (94.7)	477 (40.7)	695 (59.3)	297 (25.3)	875 (74.7)
Substance use disorder diagnosis [c]					
Yes, n (%)	280 (22.6)	140 (50.0)	140 (50.0)	89 (31.8)	191 (68.2)
No, n (%)	957 (77.4)	366 (38.2)	591 (61.8)	223 (23.3)	734 (76.7)
Suicidal ideation diagnosis [c]					
Yes, n (%)	46 (3.7)	34 (73.9)	12 (26.1)	22 (47.8)	24 (52.2)
No, n (%)	1191 (96.3)	472 (39.6)	719 (60.4)	290 (24.3)	901 (75.7)
Veteran utilization assessed in past 12 months					
Any Veteran VA non-PTSD mental health visit [c]					
Yes, n (%)	708 (57.2)	320 (45.2)	388 (54.8)	197 (27.8)	511 (72.2)
No, n (%)	529 (42.8)	186 (35.2)	343 (64.8)	115 (21.7)	414 (78.3)
Any Veteran VA primary care visit [c]					
Yes, n (%)	1178 (95.2)	490 (41.6)	688 (58.4)	303 (25.7)	875 (74.3)
No, n (%)	59 (4.8)	16 (27.1)	43 (72.9)	9 (15.3)	50 (84.7)
Any Veteran VA ED/urgent care visit [c]					
Yes, n (%)	374 (30.2)	184 (49.2)	190 (50.8)	125 (33.4)	249 (66.6)
No, n (%)	863 (69.8)	322 (37.3)	541 (62.7)	187 (21.7)	676 (78.3)
Any Veteran VA conjoint therapy visit [c]					
Yes, n (%)	230 (18.6)	116 (50.4)	114 (49.6)	83 (36.1)	147 (63.9)
No, n (%)	1007 (81.4)	390 (38.7)	617 (61.3)	229 (22.7)	778 (77.3)

SD = Standard deviation, No. = number, CESD-10 = Center for Epidemiological Studies - Depression Scale – 10, PCAFC = Program of Comprehensive Assistance for Family Caregivers, CSP = Caregiver Support Program, PTSD = post-traumatic stress disorder, ED = emergency department

The term family member is used, but people in this group could be family members or friends.

Treatment initiation defined as having participated in at least one session during the follow up period; completion of an adequate dose defined as having participated in at least 8 sessions within 6 months during the follow up period

[a] Obtained via responses from caregiver survey

[b] Obtained via responses from caregiver survey; supplemented with electronic health record data if item response in survey was missing

[c] Obtained via electronic health record

[d] Obtained from administrative records of the Caregiver Support Program

[e] Missing data: Family member gender (2), family member age (131), Veteran race (21), Veteran Hispanic/Latino(a) ethnicity (7), family member race (86), family member Hispanic/Latino(a) ethnicity (26), people living in household (47), persons in household under 18 (61), family member education (31), Veteran and family member partnered (4), Veteran marital status (3), family member marital status (17), Veteran's financial situation (186), family member financial situation (157), family member perceived financial situation score (103), family member employment status (182), average no. of days family member provides for Veteran (107), family member health status (177), Zarit score (221), CESD-10 score (272), Positive aspects of caregiving score (230), family member missed doctor's appointments (207), family member flu shot (212), drive time to nearest VA primary care (3), drive time to nearest VA secondary care facility (6), drive time to nearest VA tertiary care facility (14), Used Caregiver Support Line (237), CSP helped caregiver to assist Veteran to be more engaged in health care (191), CSP has helped family member to support progress in my Veteran's rehabilitation or treatment (191), Veteran service connection (1), how would you rate his/her emotional problems (258)

[f] Includes Asian, American Indian or Alaska Native, Native Hawaiian or Other Pacific Islander, or multiple racial categories

[g] The VA Nosos risk score predicts health care costs using demographic data, physical and mental health diagnoses, prescription data, and health care utilization from the current fiscal year (FY 2015). The Nosos score is centered at 1; a score of 1 indicates that the Veteran has costs that are the national average for VA patients while a score of 1.5 indicates that the expected costs for that patient are 1.5 times higher than the average VA patient.[1]

References for validated survey measures: Family member health status (VR-12) [2]; Subjective Zarit burden score [3]; Positive aspects of caregiving [4]; CESD-10 score [5]; Caregiver Reaction Assessment subscale on perceived financial strain [6].

References

1. Wagner TH, Upadhyay A, Cowgill E, et al. Risk Adjustment Tools for Learning Health Systems: A Comparison of DxCG and CMS-HCC V21. Health Serv Res 2016;51:2002-19.
2. Jones D, Kazis L, Lee A, et al. Health status assessments using the Veterans SF-12 and SF-36: methods for evaluating otucomes in the Veterans Health Administration. J Ambul Care Manage 2001;24:68-86.
3. Bedard M, Molloy DW, Squire L, Dubois S, Lever JA, O'Donnell M. The Zarit Burden Interview: a new short version and screening version. Gerontologist 2001;41:652-7.
4. Tarlow BJ, Wisniewski SR, Belle SH, Rubert M, Ory MG, Gallagher-Thompson D. Positive Aspects of Caregiving. Res Aging 2016;26:429-53.
5. Andresen EM, Malmgren JA, Carter WB, Patrick DL. Screening for depression in well older adults: evaluation of a short form of the CES-D (Center for Epidemiologic Studies Depression Scale). Am J Prev Med 1994;10:77-84.
6. Given CW, Given B, Stommel M, Collins C, King S, Franklin S. The caregiver reaction assessment (CRA) for caregivers to persons with chronic physical and mental impairments. Res Nurs Health 1992;15:271-83.
