# Supplementary material for: Exploring the importance of predisposing, enabling, and need factors for promoting Veteran engagement in mental health therapy for post-traumatic stress: a multiple methods study
Source: BMC Psychiatry. 2023 May 27;23:372. doi: 10.1186/s12888-023-04840-7 (PMC10219808; doi:10.1186/s12888-023-04840-7)
Supplement: Supplementary file 3 — Additional file 3. Script: Family member of Veteran in treatment. This script was administered to family members of Veterans who were interviewed prior to starting mental health therapy for PTSD. [file 12888_2023_4840_MOESM3_ESM.docx]

**GO VA Families** (**G**etting **O**ur **V**eteran **A**ccess to MH services through enabling resources and **FAMILY** support)

**Caregiver Qualitative Interview Script**

*Aim 1b: How do Veteran and family enabling factors (e.g. financial strain, family strain, lack of employment, logistical barriers) influence use of PTSD psychotherapy?*

*Note to IRB: This is an in-depth qualitative interview guide.*

*Exact order and working of questions may vary.*

Study ID#:

Names:

Telephone number:

Address:

Hello, this is [Name of Study staff]. I am calling from the VA Medical Center in Durham North Carolina regarding the **GO VA Families** Study. **May I speak with [Name of Caregiver] regarding an interview that [Name of Study Staff] previously scheduled with you?**

*****No:**

Thank you, is there a better time to call back?

****Voicemail:**

“Hello. This message is for [Name of Caregiver]. My name is [Name of Study staff], and I am calling from the VA Medical Center in Durham North Carolina regarding the **GO VA Families** study. Please call me at (919) 286-0411, extension 175196. Our Toll-Free number is 1-888-878-6890, extension 175196. Thank you and I look forward to speaking with you.”

***Yes:**

****Study Synopsis:**

“Thanks so much again for taking the time to speak with me! I’m just going to briefly go over with you some of the information that you and Breana talked about and make sure that you don’t have any questions. Does that sound good?

Great! So, as you might remember, the GO VA Families Study is a national study to help us understand how to improve Veterans’ experience of treatment for traumatic stress and how their family may be able to help. In order to learn more about this, we are speaking with both veterans and family members about their thoughts about therapy and care associated with trauma from the stress of military service. That is what we’re going to be talking about today.

The questions I have for you today will probably take about an hour, but if you need to stop at any time, just let me know. This interview is completely voluntary, so that means if you want to skip a question, just let me know, and we can move on to the next one. You can also end the interview at any time.

And I wanted to remind you, there are no right or wrong answers. We want to hear about your experiences, so feel free to answer freely, as your name will not be associated with anything you tell us today. The information that you provide in the interview will not be shared with any other party.

Do you have any questions about any of that?

Ok, great! I would like to audio record this interview, so we can make sure we remember what you tell us today. Do you agree to allow us to audio-record this interview? Please let me know if at any time you would like me to stop recording.

Do you have any other questions before we start?”

<<start recording here>> Note that consent does not need to be audio recorded.

**Directions to Interviewer:** Inform family member that interview will begin with broad questions about them and their perspective of care for traumatic stress and then move to questions about specific factors that may have played a role in their perspectives.

- 1. Would you please confirm how you are related to [Veteran]? How would you like me to refer to [him/her] during the interview?
  2. Let’s start by you telling me a little bit about yourself. *Probe for things like significant other, children, current employment, caregiver veteran status etc.*

Thank you for sharing that information with me. Now, I’d like to talk to you more about treatment for [veteran name] post-traumatic stress disorder.

1. Tell me about how you came to learn that the Veteran had been diagnosed with post-traumatic stress.
2. Tell me about your understanding of the referral for treatment for post-traumatic stress that [Veteran name] received in [orient them to the month of this referral].

*Probe to characterize past treatment attempts:*

- 1. Can you talk about the Veteran’s past attempts at treatment, if any?
  2. How many times they have attempted treatment? For what dx?
  3. Was your veteran successful at completing that course of treatment?

1. What is your understanding of what would be involved in treatment for post-traumatic stress?
2. Do you think that treatment would be helpful for your loved one? *(by “helpful” we mean: might it reduce PTS symptoms, help Veteran to make progress and/or improve interactions with other people in his/her life?)*
   1. Why or why not?
   2. *Probe for specific ways that the family member through it would or would not be helpful to them [i.e. changes in symptoms, family function, ability to participate in other activities, engage in work, engage in family life, etc.]*
3. Veterans have different reasons for not following through with referrals for treatment for post-traumatic stress. What are some reasons you believe that [Veteran’s name] did not attend the referral appointment?
   1. Tell me about that.
      1. ***Probe for details re enabling factors as needed*** *(e.g. financial trouble that is not related to paying for the appointment, caring for dependents, life chaos, s/he doesn’t believe that the treatment will work, treatment will result in bad outcomes, s/he is not ready, h/she doesn’t need it, stigma, etc.)*
      2. **If not mentioned**: Sometimes practical things (e.g., transportation, scheduling, employment, home stability) make it difficult to attend treatment. Did any practical barriers affect [Veteran’s name] ability to attend the appointment?
   2. Thanks for sharing those reasons with me. Are there any other reasons you think impacted his/her ability to attend the appointment?

Now I’d like to talk a little bit more about how you feel you might or might not be involved in the Veteran’s care and treatment for post-traumatic stress.

1. In what ways, if at all, do you think you should be involved in your loved one’s treatment?
   1. **If involvement is mentioned****:** How have you been involved in the past? What might your involvement look like? How might your involvement be helpful?
      1. *Probe for whether the family member perceives that s/he could provide social support (i.e. encouragement), practical support (i.e. driving, waiting, making/organizing appointments, reminders), or something else.*
      2. Could you describe any ways that the Veteran might perceive your involvement to be not helpful?
   2. **If involvement is not mentioned**: Tell me more about how you’ve come to that decision
2. Do you think the Veteran should engage in treatment for post-traumatic stress in the future? Why or why not?
3. Is [Veteran name] seeking any mental health care for traumatic stress outside of the VA?
   1. **If yes**, can you tell me about [his/her] experiences seeking this care?
   2. **If yes,** what do you think some of the reasons are that [he/she] decided to seek this care?
   3. **If no,** to your knowledge, has [he/she] considered seeking mental health care for traumatic stress?
      1. **If yes**, can you tell me a little bit about that?

I want to sincerely thank you for your time and for the helpful information that you have provided. If you think of anything else to add or share about these topics later, please feel free to call the principal investigator of this project Dr. Megan Shepherd-Banigan at 919-286-0411 ext. 175196.

We will send you a check for $25 in appreciation for your time. We will process your payment information this week, but it may take up to 4-6 weeks for you to receive the check.

Again, thank you for your time and especially for all you do to support the Veteran.
