# Supplementary material for: Exploring the importance of predisposing, enabling, and need factors for promoting Veteran engagement in mental health therapy for post-traumatic stress: a multiple methods study
Source: BMC Psychiatry. 2023 May 27;23:372. doi: 10.1186/s12888-023-04840-7 (PMC10219808; doi:10.1186/s12888-023-04840-7)
Supplement: Supplementary file 4 — Additional file 4. Script: Family member of Veteran prior to treatment. This script was administered to family members of Veterans who were interviewed while they were in therapy for PTSD. [file 12888_2023_4840_MOESM4_ESM.docx]

**GO VA Families** (**G**etting **O**ur **V**eteran **A**ccess to MH services through enabling resources and **FAMILY** support)

**Caregiver Qualitative Interview Script**

*Aim 1b: How do Veteran and family enabling factors (e.g. financial strain, family strain, lack of employment, logistical barriers) influence use of PTSD psychotherapy?*

*Note to IRB: This is an in-depth qualitative interview guide.*

*Exact order and working of questions may vary.*

Names:

Telephone number:

Address:

Hello, this is [Name of Study Staff]. I am calling from the VA Medical Center in Durham North Carolina regarding the **GO VA Families** Study. **May I speak with [Name of Caregiver] regarding an interview that [Name of Study Staff] previously scheduled with you?**

*****No:**

Thank you, is there a better time to call back?

****Voicemail:**

“Hello. This message is for [Name of Caregiver]. My name is [Name of Study staff], and I am calling from the VA Medical Center in Durham North Carolina regarding the **GO VA Families** study. Please call me at (919) 286-0411, extension 175196. Our Toll-Free number is 1-888-878-6890, extension 175196. Thank you and I look forward to speaking with you.”

***Yes:**

Is this still a good time to interview you for the GO VA Families study? We recognize that this is a unique time and are all having to manage so many different things now with the Corona virus (COVID-19).

**If yes:** Ok great. I want to review a few things before we begin.

****Study Synopsis:**

“Thanks so much again for taking the time to speak with me! I’m just going to briefly go over with you some of the information that you and [Name of Study Staff] talked about and make sure that you don’t have any questions. Does that sound good?

Great! So, as you might remember, the ***GO VA Families Study*** is a national study to help us understand how to improve Veterans’ experience of treatment for posttraumatic stress disorder and how their family may be able to help. In order to learn more about this, we are speaking with both veterans and family members about their thoughts about therapy and care associated with trauma from the stress of military service.  That is what we’re going to be talking about today.

The questions I have for you today will probably take about an hour, but if you need to stop at any time, just let me know. This interview is completely voluntary, so that means if you want to skip a question, just let me know, and we can move on to the next one. You can also end the interview at any time.

And I wanted to remind you, there are no right or wrong answers.  We want to hear about your experiences, so feel free to answer freely, as your name will not be associated with anything you tell us today.   The information that you provide in the interview will not be shared with any other party.

Do you have any questions about any of that?

Ok, great! I would like to audio record this interview, so we can make sure we remember what you tell us today. Do you agree to allow us to audio-record this interview?  Please let me know if at any time you would like me to stop recording.

Do you have any other questions before we start?”

<<start recording here>> Note that consent does not need to be audio recorded.

**Directions to Interviewer:** Inform family member that interview will begin with broad questions about them and their perspective of PTSD treatment and then move to questions about specific factors that may have played a role in their perspectives.

1. Would you please confirm how you are related to [Veteran]? How would you like me to refer to [him/her] during the interview?
2. Let’s start by you telling me a little bit about yourself.

*Probe for things like significant other, children, current employment, veteran status of caregiver etc.*

Thank you for sharing that information with me. Now, I’d like to talk to you more about [role of Veteran to caregiver/][Veteran’s name] PTSD diagnosis and treatment.

1. Tell me about how you came to learn that [Veteran’s name] had been diagnosed with post-traumatic stress.
2. I understand that [Veteran’s name] has recently attended treatment for post-traumatic stress. Tell me whether s/he has completed his/her participation in this treatment?
   1. **If yes:** do you know whether s/he finished all the expected sessions?
   2. **If no:** do you know whether s/he plans to attend another session?

*Probe to characterize past treatment attempts:*

- 1. Can you talk about the Veteran’s past attempts at treatment, if any?
  2. How many times they have attempted treatment? For what dx?
  3. Was your veteran successful at completing that course of treatment?

1. Do you think treatment for post-traumatic stress has been helpful for [Veteran’s name]? (by helpful we mean: might reduce PTSD symptoms, help Veteran to make progress and/or improve interactions with other people in his/her life)
   1. In what ways do you think treatment for post-traumatic stress has been helpful for [Veteran’s name]?
   2. *Probe for specific ways that the family member thinks it has been or has not be helpful to them [i.e. changes in symptoms, family function, ability to participate in other activities, engage in work, helped Veteran to make progress, engage in family life, etc.]*
2. Are there ways in which the treatment for post-traumatic stress has not been helpful for [Veteran’s name]?
   1. *Probe for specific ways that the family members thinks treatment would not be helpful to Veteran [i.e. worsen symptoms, make him/her do things that feel frightening*
3. Veterans have different reasons for following through with treatment for post-traumatic stress. What are some reasons you believe that the Veteran attended these appointments?
   1. *Probe for details re enabling factors as needed* *(e.g. encouragement from family member/other friends, desire to improve, belief that treatment would help, availability to attend appointments, strong bond with therapist, etc.)*
   2. Were there any factors that made it difficult for [Veteran’s name] to attend the appointments? *This will be especially important for veterans who dropped out;* *probe for details about challenges; including financial trouble, caring for dependents, life chaos, worsening of symptoms/treatment was difficult, challenges with therapy, transportation difficulties, difficulty scheduling, employment which made it hard to get to the appointments, etc.*
   3. Thanks for sharing those reasons with me. Are there any other reasons you think impacted his/her ability to attend the appointment?

Now I’d like to talk a little bit more about how you feel you have or have not been involved in the Veteran’s treatment for post-traumatic stress.

1. Can you describe any ways in which you have been involved in [Veteran’s name] treatment for post-traumatic stress?
   1. *If no*: can you tell me a little bit about why you haven’t been involved? *Probe for: Veteran didn’t want family member involved, family member didn’t have time, etc.*
   2. *If yes*: Can you describe how your involvement has been beneficial?

*Probe for whether the family member perceives that s/he could provide social support (i.e. encouragement), practical support (i.e. driving, waiting, making/organizing appointments, reminders), or something else.*

- 1. *If yes*: are there ways in which you or the Veteran might want to change how you have been involved?

1. Did you encourage [Veteran’s name] to pursue treatment for post-traumatic stress?
   1. **If yes**: Can you describe to me what that conversation looked like?

- How did [Veteran’s name] respond?

**If no**: Can you tell me why not?

1. Do you think the Veteran should continue to engage in treatment for post-traumatic stress in the future? Why or why not?
2. Can you describe to me the type of support you might need to help the Veteran engage in treatment for post-traumatic stress? Is there support that you have already received that was not helpful?
3. Is [Veteran name] seeking any mental health care for traumatic stress outside of the VA?
4. **If yes**, can you tell me about [his/her] experiences seeking this care?
5. **If yes**, what do you think some of the reasons are that [he/she] decided to seek this care?
6. **If no**, to your knowledge, has [he/she] considered seeking mental health care for traumatic stress?
   1. **If yes**, can you tell me a little bit about that?
7. We recognize that these are unique times to be asking you about [Veteran’s name] mental health care. How much do you think the Coronavirus (COVID-19) outbreak has affected your answers today? Please choose the response option that is the best match for you.
   1. To an extremely large extent
   2. To a large extent
   3. To a moderate extent
   4. To a small extent
   5. Not at all
   6. Don’t know
8. Please tell me a little more about your answer.
9. Related to that, how much do you think the Coronavirus (COVID-19) outbreak has changed your perception about [Veteran’s name] ability to seek mental health care? Tell me more about your perspective.
10. How much do you think the Coronavirus (COVID-19) outbreak has changed your ability to support [Veteran’s name] in care and treatment for post-traumatic stress? In what ways?

I want to sincerely thank you for your time and for the helpful information that you have provided. If you think of anything else to add or share about these topics later, please feel free to call the principal investigator of this project Dr. Megan Shepherd-Banigan at 919-286-0411 ext. 175196.

We will send you a check for $25 in appreciation for your time. We will process your payment information this week, but it may take up to 4-6 weeks for you to receive the check.

Again, thank you for your time and especially for all you do to support the Veteran.
