# Supplementary material for: Exploring the importance of predisposing, enabling, and need factors for promoting Veteran engagement in mental health therapy for post-traumatic stress: a multiple methods study
Source: BMC Psychiatry. 2023 May 27;23:372. doi: 10.1186/s12888-023-04840-7 (PMC10219808; doi:10.1186/s12888-023-04840-7)
Supplement: Supplementary file 5 — Additional file 5. Script: Veteran in treatment. This script was administered to Veterans who were interviewed while they were in therapy for PTSD. [file 12888_2023_4840_MOESM5_ESM.docx]

**GO VA Families** (**G**etting **O**ur **V**eteran **A**ccess to MH services through enabling resources and **FAMILY** support)

**Veteran Qualitative Interview Script**

*Note to IRB: This is an in-depth qualitative interview guide.*

*Exact order and working of questions may vary.*

Names:

Telephone number:

Address:

Hello, this is [Name of Study staff]. I am calling from the VA Medical Center in Durham North Carolina regarding the **GO VA Families** Study. **May I speak with [Name of Veteran] regarding an interview that [Name of Study Staff] previously scheduled with you?**

*****No:**

Thank you, is there a better time to call back?

****Voicemail:**

“Hello. This message is for [Name of Caregiver]. My name is [Name of Study staff], and I am calling from the VA Medical Center in Durham North Carolina regarding the **GO VA Families**  study. Please call me at (919) 286-0411, extension 175196. Our Toll-Free number is 1-888-878-6890, extension 175196. Thank you and I look forward to speaking with you.”

***Yes:**

Is this still a good time to interview you for the GO VA Families study? We recognize that this is a unique time and are all having to manage so many different things now with the Cororona virus (COVID-19).

**If yes:** Ok great. I want to review a few points before we begin.

****Study Synopsis:**

“Thanks so much again for taking the time to speak with me! I’m just going to briefly go over with you some of the information that you and [Name of Study Staff] talked about and make sure that you don’t have any questions. Does that sound good?

Great! So as you might remember, the GO VA Families Study is a national study to help us understand how to improve Veterans’ experience of treatment for posttraumatic stress disorder and how their family may be able to help. In order to learn more about this, we are speaking with both veterans and family members about their thoughts about treatment associated with trauma from the stress of military service. That is what we’re going to be talking about today.

The questions I have for you today will probably take about an hour, but if you need to stop at any time, just let me know. This interview is completely voluntary, so that means if you want to skip a question, just let me know, and we can move on to the next one. You can also end the interview at any time. The information that you provide in the interview will not be shared with any other party.

And I wanted to remind you, there are no right or wrong answers. We just want to hear about your experiences, so feel free to answer freely, as your name will not be associated with anything you tell us today.

Do you have any questions about any of that?

Ok, great! I would like to audio record this interview, so we can make sure we remember what you tell us today. Do you agree to allow us to audio-record this interview? Please let me know if at any time you would like me to stop recording.

Do you have any other questions before we start?”

<<start recording here>> Note that consent does not need to be audio recorded.

**Directions to Interviewer:** Inform Veteran interview will begin with broad questions about them and their level of interest in treatment for military-service related stress—or PTSD—and then move to questions about specific factors that may have played a role in their level of interest, including social support.

1. Let’s start by you telling me a little bit about yourself like where you work, whether you are married, etc.
   1. *Probe for information about children, hobbies, etc.*

Thanks so much for that information. I’d like to talk a little bit more now about post-traumatic stress disorder and your involvement in treatment.

1. Can you tell me about how you came to learn that you have post-traumatic stress?
2. Have you received treatment for this in the past; before the treatment that you are currently receiving? Tell me about that. *[note to reviewer: it is possible that the veteran had received prior mental health treatment for other conditions such as anxiety or depression]*
   1. What made you pursue treatment at that time?
   2. **If they say a referral was made but did not follow through:** What got in the way of your pursuing treatment at that time?

*Probe to characterize past treatment attempts:*

- 1. About how many times you have attempted treatment? For what dx?
  2. Were you successful at completing that course of treatment?

1. My understanding is that you were referred for mental health therapy for treatment for mliary service-related stress within the past 12 months and that you have attended at least one treatment visit. Is this correct?
   1. **If yes:** when was the last visit that you had? Do you plan to attend another one in the next month? Do you recall how many visiits you have attended?
   2. **If no:** *use Veteran_priortx document*
2. Do you recall which provider gave you a referral for the treatment for for military service-related stress that you are currently/recently using?
   1. **If yes:** could you describe the conversation that you and your provider had about this treatment?
3. Do you think treatment for post-traumatic stress has been helpful to you at this time?
   1. In what ways do you think that PTS treatment has changed your life (by helpful we mean: it might reduce PTSD symptoms, help you to make progress and/or improve interactions with other people in our life)
      1. *Probe for specific ways that the Veteran thought it would or would not be helpful to them [i.e. changes in symptoms, in family functioning, in ability to participate in activities that are meaningful/fulfilling, engage in work, engage in family life, etc.].*
4. Are there ways in which treatment has not been helpful for you?
5. Veterans have different reasons for following through or not following through with treatment for posttraumatic stress. What are some reasons you attended your mental health visits?
   1. *Probe for details re enabling factors as needed (e.g. encouragement from family member/other friends, desire to improve, belief that treatment would help, availability to attend appointments, strong bond with therapist, etc.)*
   2. **Please describe any factors that made it difficult for you to attend these appoinments.** *This will be especially important for veterans who dropped out;* *probe for details about challenges; including financial trouble, caring for dependents, life chaos, worsening of symptoms/treatment was difficult, challenges with therapy, transportation difficulties, difficulty scheduling, employment which made it hard to get to the appointments, etc.*
   3. Thanks for sharing all of those. Are there any other reasons that kept you from attending the appointment?

Now I’d like to talk a little bit more about your support systems and how those may relate to treatment

for for military service-related stress.

1. Who in your life would you consider to be your social support?
   1. **If names people other than those in Question 1:** Earlier, you mentioned [name people from Question 1]. Do you consider them social support? How does/do [person/people from Question 1] show their support?
2. Did any of these people know you had been referred for treatment for military service-related stress?
   1. **If yes:** What did they think about that?
   2. **If no:** Why is that?
3. Did their opinion influence your decision to move forward with this treatment?
   1. Why or why not?
   2. Can you tell me about converations that you have had with [name of family member] about mental health treatment? How did they go? How did they make you feel?
      1. *Probe for whether these conversations felt supportive of the Veteran’s wishes/treatment goals and encouraged the Veteran to want to get into treatment, whether they enabled the Veteran to continue not seeking help, or whether they felt unhelpful or discouraging.*
4. Can you tell me about other ways that [names of people] have or have not supported you during this treatment?
5. What are your thoughts about involving people in your life in your treatment for post-traumatic stress?
   1. How has it been helpful or not?
   2. What does helpful involvement from [name of family member] look like?
   3. What gets in the way of [name of family member] being involved in your treatment?
      1. *Probe for issues such as Veteran unwillingness, lack of knowledge of VA system, work or family related barriers.*
   4. Can you describe to me what type of support you and [name of family member] could use to help you engagement in treatment for post-traumatic stress? Is there support that you have already received that was or was not helpful?
6. Do you intend to continue to attending your these treatment visits in the future?
   1. Why or why not?
7. Are you also seeking any mental health care for yourself, outside of the VA?
8. **if yes**, can you tell me about your experiences seeking this care for yourself?
9. **If yes**, what are some of the reasons you decided to seek this care?
10. **If no**, have you considered seeking MH care?
    1. **If yes**: Can you tell me a little bit about that?
11. We recognize that these are unique times to be asking you about mental health care. How much do you think the Coronavirus (COVID-19) outbreak has affected your answers today? Please choose the response option that is the best match for you.
    1. To an extremely large extent
    2. To a large extent
    3. To a moderate extent
    4. To a small extent
    5. Not at all
    6. Don’t know
12. Please tell me a little more about your answer.
13. Related to that, how much do you think the Coronavirus (COVID-19) outbreak has changed your perception about seeking mental health care? Tell me more about your perspective.
14. How much do you think the Coronavirus (COVID-19) outbreak has changed your ability to seek care and treatment for post-traumatic stress? In what ways?

I want to sincerely thank of you for your time and for the helpful information that you have provided. If you think of anything else to add or share about these topics later, please feel free to call the principal investigator of this project Dr. Megan Shepherd-Banigan at 919-286-0411 ext. 175196.

We will send you a check for $25 in appreciation for your time. We will process your payment information this week, but it make take up to 4-6 weeks for you to receive the check. Again, thank you for your time.
