# Supplementary material for: Exploring the importance of predisposing, enabling, and need factors for promoting Veteran engagement in mental health therapy for post-traumatic stress: a multiple methods study
Source: BMC Psychiatry. 2023 May 27;23:372. doi: 10.1186/s12888-023-04840-7 (PMC10219808; doi:10.1186/s12888-023-04840-7)
Supplement: Supplementary file 6 — Additional file 6. Script: Veteran prior to treatment. This script was administered to Veterans who were interviewed prior to starting mental health therapy for PTSD. [file 12888_2023_4840_MOESM6_ESM.docx]

**GO VA Families** (**G**etting **O**ur **V**eteran **A**ccess to MH services through enabling resources and **FAMILY** support)

**Veteran Qualitative Interview Script**

*Note to IRB: This is an in-depth qualitative interview guide.*

*Exact order and wording of questions may vary.*

Study ID#:

Names:

Telephone number:

Address:

Hello, this is [Name of Study staff]. I am calling from the VA Medical Center in Durham North Carolina regarding the **GO VA Families** Study. **May I speak with [Name of Veteran] regarding an interview that [Name of Study Staff] previously scheduled with you?**

*****No:**

Thank you, is there a better time to call back?

****Voicemail:**

“Hello. This message is for [Name of Caregiver]. My name is [Name of Study staff], and I am calling from the VA Medical Center in Durham North Carolina regarding the **GO VA Families**  study. Please call me at (919) 286-0411, extension 175196. Our Toll-Free number is 1-888-878-6890, extension 175196. Thank you and I look forward to speaking with you.”

***Yes:**

Is this still a good time to interview you for the GO VA Families study? We recognize that this is a unique time and are all having to manage so many different things now with the Corona virus (COVID-19).

**If yes:** Ok great. I want to review a few things before we begin.

****Study Synopsis:**

“Thanks so much again for taking the time to speak with me! I’m just going to briefly go over with you some of the information that you and [Name of Study Staff] talked about and make sure that you don’t have any questions. Does that sound good?

Great! So as you might remember, the GO VA Families Study is a national study to help us understand how to improve Veterans’ experience of treatment for posttraumatic stress disorder and how their family may be able to help. In order to learn more about this, we are speaking with both veterans and family members about their thoughts about treatment associated with trauma from the stress of military service. That is what we’re going to be talking about today.

The questions I have for you today will probably take about an hour, but if you need to stop at any time, just let me know. This interview is completely voluntary, so that means if you want to skip a question, just let me know, and we can move on to the next one. You can also end the interview at any time.

And I wanted to remind you, there are no right or wrong answers. We want to hear about your experiences, so feel free to answer freely, as your name will not be associated with anything you tell us today. The information that you provide in the interview will not be shared with any other party.

Do you have any questions about any of that?

Ok, great! I would like to audio record this interview, so we can make sure we remember what you tell us today. Do you agree to allow us to audio-record this interview? Please let me know if at any time you would like me to stop recording.

Do you have any other questions before we start?”

<<start recording here>> Note that consent does not need to be audio recorded.

**Directions to Interviewer:** Orient Veteran to the timeline for mental health referral. Inform Veteran interview will begin with broad questions about them and their level of interest in care and treatment for military related stress—or PTSD—and then move to questions about specific factors that may have played a role in their level of interest, including social support.

1. Let’s start by you telling me a little bit about yourself like where you work, whether you are married, etc..
   1. *Probe for information about children, hobbies, etc.*

Thanks so much for that information. I’d like to talk a little bit more now about post-traumatic stress disorder? and your interest in treatment for this.

1. Tell me about how you came to learn that you have post-traumatic stress.
2. Have you received treatment for this in the past? Tell me about that.
   1. What made you pursue treatment at that time? **If they say a referral was made but did not follow through:** What got in the way of your pursuing treatment at that time?
3. My understanding is that your health care provider [Name of Provider if available] gave you referral for treatment for military service-related stress in [orient them to the month of this referral] and that you have not yet attended a treatment visit. Is this correct?
   1. **If yes:** Tell me what your provider said about that referral. **Probes:** Did s/he say why s/he was placing the referral? Did s/he speak about what kind of treatment you were being referred for? What did you think about all of that?
   2. ***If no*** *because has already attended a PTSD therapy appointment: use Veteran_intx script.*
4. What is your understanding about what would be involved in treatment for military service-related stress?
5. Do you think mental health treatment would be helpful to you at this time?
   1. Why or why not?
   2. *Probe for specific ways that the Veteran thought it would or would not be helpful to them [i.e. changes in symptoms, in family functioning, in ability to participate in activities that are meaningful/fulfilling, engage in work, engage in family life, etc.].*
6. Veterans have different reasons for following through and not following through with referrals for treatment for posttraumatic stress. What are some reasons you did not attend your referral appointment?
   1. *Probe for details re enabling factors as needed (e.g. financial trouble, caring for dependents, life chaos, I don’t believe that the treatment will work, treatment will result in bad outcomes, I am not ready, I don’t know how to make an appointment, I don’t need it)*
      1. *If still not clear: “What would it look like if you are ready?”*
   2. **If not mentioned:** Sometimes practical things (e.g., transportation, scheduling, employment, home stability) make it difficult to attend treatment. Did any practical barriers affect your ability to attend your appointment?
   3. Thanks for sharing all of those. Are there any other reasons that impacted your ability to attend the appointment?

Now I’d like to talk a little bit more about your support systems and how those may relate to care and treatment for post-traumatic stress.

1. Who in your life would you consider to be your social support?
   1. **If names people other than those in Question 1:** Earlier, you mentioned [name people from Question 1]. Do you consider them social support? How does/do [person/people from Question 1] show their support?
2. Did any of these people know you had been referred for treatment for post-traumatic stress?
   1. **If yes:** What did they think about that?
   2. **If no:** Why is that?
3. Did their opinion influence your decision to not move forward with this treatment?
4. What are your thoughts about involving people in your life in your treatment for military service-related stress?
   1. Would it be helpful? Why or why not?
5. Do you intend to engage in treatment for military service-related stress in the future?
   1. Why or why not?
6. Are you seeking any mental health care for yourself, outside of the VA?
   1. **If yes**, can you tell me about your experiences seeking this care for yourself?
   2. **If yes**, what are some of the reasons you decided to seek this care?
   3. **If no**, have you considered seeking MH care?
      1. **If yes,** Can you tell me a little bit about that?
7. We recognize that these are unique times to be asking you about mental health care. How much do you think the Coronavirus (COVID-19) outbreak has affected your answers today? Please choose the response option that is the best match for you.
   1. To an extremely large extent
   2. To a large extent
   3. To a moderate extent
   4. To a small extent
   5. Not at all
   6. Don’t know
8. Please tell me a little more about your answer.
9. Related to that, how much do you think the Coronavirus (COVID-19) outbreak has changed your perception about seeking mental healtht care? Tell me more about your perspective.
10. How much do you think the Coronavirus (COVID-19) outbreak has changed your ability to seek care and treatment for post-traumatic stress? In what ways?

I want to sincerely thank of you for your time and for the helpful information that you have provided. If you think of anything else to add or share about these topics later, please feel free to call the principal investigator of this project Dr. Megan Shepherd-Banigan at 919-286-0411 ext. 175196.

We will send you a check for $25 in appreciation for your time. We will process your payment information this week, but it make take up to 4-6 weeks for you to receive the check.

Again, thank you for your time.
